# The structure of MadC from *Clostridium maddingley* reveals new insights into class I lanthipeptide cyclases

**DOI:** 10.3389/fmicb.2022.1057217

**Published:** 2023-01-18

**Authors:** C. Vivien Knospe, Michael Kamel, Olivia Spitz, Astrid Hoeppner, Stefanie Galle, Jens Reiners, Alexej Kedrov, Sander H. J. Smits, Lutz Schmitt

**Affiliations:** ^1^Institute of Biochemistry, Heinrich Heine University Düsseldorf, Düsseldorf, Germany; ^2^Synthetic Membrane Systems, Institute of Biochemistry, Heinrich Heine University Düsseldorf, Düsseldorf, Germany; ^3^Center for Structural Studies, Heinrich Heine University Düsseldorf, Düsseldorf, Germany

**Keywords:** lanthipeptide, cyclases, X-ray structure, class I lantibiotic, ITC

## Abstract

The rapid emergence of microbial multi-resistance against antibiotics has led to intense search for alternatives. One of these alternatives are ribosomally synthesized and post-translationally modified peptides (RiPPs), especially lantibiotics. They are active in a low nanomolar range and their high stability is due to the presence of characteristic (methyl-) lanthionine rings, which makes them promising candidates as bacteriocides. However, innate resistance against lantibiotics exists in nature, emphasizing the need for artificial or tailor-made lantibiotics. Obviously, such an approach requires an in-depth mechanistic understanding of the modification enzymes, which catalyze the formation of (methyl-)lanthionine rings. Here, we determined the structure of a class I cyclase (MadC), involved in the modification of maddinglicin (MadA) *via* X-ray crystallography at a resolution of 1.7 Å, revealing new insights about the structural composition of the catalytical site. These structural features and substrate binding were analyzed by mutational analyses of the leader peptide as well as of the cyclase, shedding light into the mode of action of MadC.

## Introduction

The need for novel antimicrobial compounds is emerging since an almost exponential increase of multi-resistant bacterial strains is observed, such as the methicillin-resistant *Staphylococcus aureus* (MRSA) strains, which are widely found in hospitals. The high biological stability of lanthipeptides due to the presence of (methyl-) lanthionine rings (Bierbaum et al., 1996) and their high activity (Breukink et al., 1999; Castiglione et al., 2007, 2008) paved the way for the usage of lantibiotics in battling multidrug resistance. They form the largest sub-family among the ribosomally synthesized and post-translationally modified peptides (RiPPs; Skinnider et al., 2016) and consist of an N-terminal leader peptide (LP) and a C-terminal core peptide (CP), which carry distinct functions during the maturation process. The LP is involved in recruiting the post-translational modification enzymes (PTMs; van der Meer et al., 1994; Patton et al., 2008; Mavaro et al., 2011; Abts et al., 2013), the export (Lagedroste et al., 2021) and in keeping the peptide in an inactive state (van der Meer et al., 1994), whereas the maturation process itself only takes place in the CP. The characteristic feature of the CP is the presence of multiple lanthionine (Lan) or methyl-lanthionine rings (Me-Lan; Kellner et al., 1988; Bierbaum, 1993; van de Kamp et al., 1995; Bierbaum et al., 1996), which are installed by a universal two-step post-translational modification process, including a dehydration step and a cyclization step. This process can be catalyzed by one enzyme (class II–IV; LanM/LanCK/LanL; Goto et al., 2010; Meindl et al., 2010; Muller et al., 2011; Dong et al., 2015), two enzymes (class I; LanC and LanB; Karakas Sen et al., 1999; Koponen et al., 2002); or even three enzymes as in class V, e.g., lexapeptide (LanK, LanX, and LanY; Xu et al., 2020).

First, dehydration of serine and threonine residues to the dehydrated amino acids 2,3-didehydroalanine (Dha) and 2,3-didehydrobutyrine (Dhb) is catalyzed by the dehydratase LanB or dehydratase domains of class II–IV enzymes (Gutowskieckel et al., 1994; Peschel et al., 1996; Karakas Sen et al., 1999; Chatterjee et al., 2005; Goto et al., 2010, 2011; Meindl et al., 2010). Afterwards, the cyclization of cysteine residues and the previously dehydrated amino acids to (Me-)Lan rings is catalyzed by LanC or the cyclization domains of class II – IV *via* a proposed Michael-type condensation mechanism. For the recently discovered class V lanthipeptides the mechanism or the modifying enzymes have not been intensively investigated so far. However, it is predicted that a three-component lanthionine synthetase is responsible for lanthionine-like modifications *via* phosphorylation and Michael-type reaction, which resembles the reactions catalyzed by class II LanM enzymes even though no homology to class II lantibiotics dehydratase and cyclase was identified (Román-Hurtado et al., 2021; Pei et al., 2022). Beside these two common modifications, which classify lanthipeptides, several other modifications such as labionin (Lab) formation (Meindl et al., 2010; Iorio et al., 2014), N,N-dimethyl lanthionine (NMe_2_Lan) ring formation (cacaoidin – class V; Román-Hurtado et al., 2021) or tailoring reactions have been discovered (Mortvedt et al., 1991; Kupke et al., 1992; van de Kamp et al., 1995; Castiglione et al., 2008; He et al., 2008; Velasquez et al., 2011; Huang and Yousef, 2015).

In this study, we focused on class I cyclases (LanC), which catalyze the nucleophilic attack of a thiol group (cysteine) towards the previously dehydrated amino acids Dha or Dhb yielding Lan or Me-Lan rings, respectively. Although, the lanthionine ring formation can occur at basic pH values (pH > 7.5) without LanC, the enzyme is still necessary for the correct ring topology by facilitating the regio-and stereoselectivity of the catalyzed reaction (Tang and van der Donk, 2013). Until now, the only known structure of a LanC for class I lanthipeptides is NisC from *L. lactis* (PDB: 2G02; 2G0D; Li et al., 2006), which revealed an α,α-toroid structure, each consisting of 7 α-helices, a coordinated zinc ion and a SH2-like domain, which is predicted to support substrate binding. Computational docking studies with nisin revealed the importance of the conserved Asp^141^ and His^212^ residues near the catalytical zinc ion for a proposed acid–base chemistry (Li and van der Donk, 2007). Furthermore, three amino acids (Cys^284^, Cys^330^, and His^331^) are ligands of a zinc ion in the center of the NisC structure, which is proposed to activate the thiol group of the cysteine of the lanthipeptide during the maturation process. The modifying enzymes of almost all classes, namely LanC, LanM, and LanL, share these conserved amino acids for binding the catalytical zinc ion, which are essential for the cyclization process as demonstrated by mutational studies on NisC (Li and van der Donk, 2007) and SgbL (Hegemann and Roderich, 2021). Beside NisC, the structure of mammalian homologs known as LanCLs [LanCL-1 (Zhang et al., 2009), LanCL-2 (Lai et al., 2021)] were solved, which also revealed an α,α-toroid structure and conservation of amino acids important for activity (relevant for zinc ion binding and protonation), and they are also capable of facilitating addition of thiolates onto dehydroamino acids even though they show no involvement in lanthionine biosynthesis in mammals (He et al., 2017; Lai et al., 2021).

However, the structure of the substrate-bound state of LanC or a cyclization domain is still unknown and the few determined structures of modification enzymes in a bound state [such as CylM (Dong et al., 2015)] did not revealed further insights of substrate binding with respect to the cyclization domain. The only other determined structure of a cyclization domain is CylM, a class II synthetase, which revealed instead of a SH2-like domain (as in NisC) another structural feature, which is assumed to be important for substrate binding or specificity. This is based on the high resemblance to a similar anti-parallel β-strand element found in substrate binding of class I LanB (NisB) and of other enzymes involved in RiPPs biosynthesis (LynD; Dong et al., 2015; Ortega et al., 2015). Unfortunately, structures of the other classes (III and IV) are not available for a comparison of their structural features and composition.

Even in the absence of structures, it was discovered that the LP of a lanthipeptide possess a crucial role in recognition by the corresponding modification enzymes due to studies regarding the binding affinity of modification enzymes to their substrates. In the case of the class I lanthipeptide nisin, the cyclase NisC displayed a K_D_ value of approximately 2 μM for all full-length precursor peptides (unmodified, dehydrated and modified nisin) independent of the maturation state of the core peptide as determined by ITC studies (Abts et al., 2013). This implies a higher relevance of the LP compared to the CP. The LP of NisA (NisL) showed a similar affinity with a K_D_ of 3.8 ± 0.6 μM. In contrast, the dehydratase NisB, which forms a complex with NisC during the maturation process (Reiners et al., 2017), showed different binding affinities dependent on the maturation state, which varied from 1 μM for unmodified prenisin and 0.3 μM for dehydrated prenisin to 10.5 μM for modified prenisin (Mavaro et al., 2011).

In comparison, the affinity of a LanM enzyme (HalM2) regarding LP, CP and full-length peptide was determined *via* fluorescence polarization (FP) studies (Thibodeaux et al., 2015). HalM2 had similar affinities regarding the fluorescently-labeled HalA2 and HalA2LP (K_D_ = 1.9 ± 0.5 μM and 3.7 ± 1.3 μM) similar to NisC for prenisin and the isolated LP, and also indicate that the leader peptide is mainly responsible for substrate recognition and binding. In contrast, the CP of HalA2 had a weak affinity (K_D_ > 500 μM), which emphasizes the importance of the LP. Furthermore, the fluorescently-labeled, fully modified HalA2 revealed an approximately 5-fold decrease of affinity (K_D_ = 10.4 ± 1.7 μM) compared to the unmodified HalA2, which resembles the properties of NisB regarding the correlation of modification state and binding affinity, which is in clear contrast to NisC, where such a correlation was not observed.

In this study, we focused on a lanthipeptide called maddinglicin (MadA) found in the genome of *Clostridium* sp. *maddingley*, which was isolated from a coal-seam formation water sample in Maddingley (Australia) in 2013 (Rosewarne et al., 2013). Three years later van Heel et al. classified maddinglicin as a class I lanthipeptide during genome mining studies (van Heel et al., 2016). In comparison to nisin, which contains five cysteines, maddinglicin contains seven cysteines, which is predicted to result in 4 intertwined rings at the C-terminus (Supplementary Figure S1A). Comparison of the N-terminus of both lanthipeptides, MadA, and NisA, reveals a high similarity of the first three rings at the N-terminus, which possess the same position within the core peptide and the same size (except for the third ring which is one amino acid (aa) smaller in MadA than in NisA; Supplementary Figures S1A, B). This led to the assumption that the first two rings have the same functionality as in nisin, i.e., binding of lipid II (Breukink et al., 1999; Hsu et al., 2004). Compared to the nisin gene cluster, the gene cluster of maddinglicin contains all components for maturation, immunity and regulation except of LanI (Supplementary Figure S1A), which leads to the assumption that the activity of maddinglicin does not entail pore formation as in the case of nisin. Additionally, the important hinge-region of nisin for pore-formation seems to be missing in maddinglicin (Supplementary Figure S1A), which emphasizes that maddinglicin will probably not form pores. Because of the unique ring formation in MadA we focused on the corresponding cyclase MadC. Here, we present the high-resolution crystal structure and biochemical characterization of MadC shedding light on the interaction of this cyclase with its natural substrate.

## Results

### Cloning, expression and purification of the lanthionine cyclase MadC

**Figure 1:**
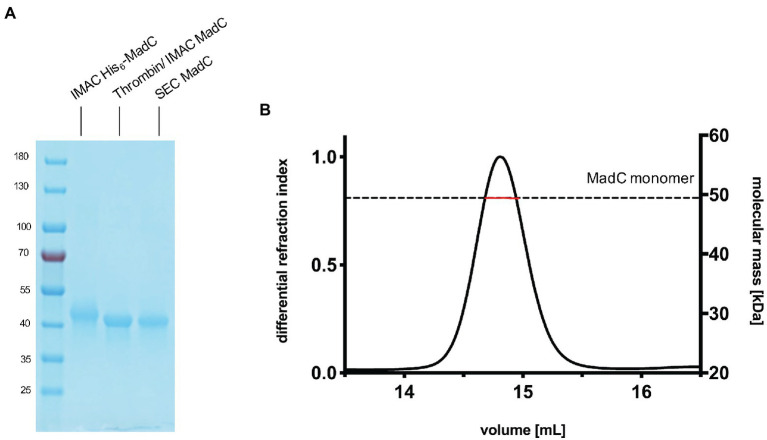
Purification of the cyclase MadC from *Clostridium maddingley*. **(A)** SDS-PAGE of samples taken during the purification of MadC. Marker: PageRuler Prestained Protein Ladder (Thermo Fisher Scientific), molecular weights in kDa are indicated. **(B)** SEC-MALS chromatogram of purified MadC (Superdex 200 Increase 10/300) in SEC Buffer 50 mM HEPES, 200 mM NaCl pH 8.

We cloned the *madC* gene of *Clostridium* sp. *maddingley* (synthetic gene ordered from GenScript) *via* Gibson assembly into a pET28b(+)-vector. This construct resulted in a hexa-histidine tagged cyclase (His_6_-MadC) containing a thrombin cleavage site at the N-terminus. *E. coli* BL21(DE3) cells were transformed with the resulting plasmid and protein expression occurred under leaky promoter condition. His_6_-MadC was purified from the cell lysate using cobalt ion affinity chromatography (see Figure 1A). The N-terminal His-Tag was cleaved off using a commercial Thrombin Cleavage-Kit. After separating cleaved MadC from His_6_-MadC using a second cobalt ion affinity chromatography step (see Figure 1A), size exclusion chromatography (SEC) was performed for further purification. The purification led to a homogenous peak in the SEC and a highly pure protein as demonstrated by SDS-PAGE (see Figure 1A). The yield of this purification amounted to approximately 20 mg of purified protein from a 2 L expression volume (around 10 g of wet cells). For the determination of the oligomeric state, SEC coupled to multi angle light scattering (SEC-MALS) was used and revealed that MadC is a monomer in solution with a molecular weight of 49.44 kDa ± 0.05 kDa, which is close to the theoretically calculated molecular weight of 49.71 kDa based on the sequence (see Figure 1B). To investigate the homogeneity in solution and to obtain an overall envelop of MadC, we performed small angle X-ray scattering (SAXS) analysis, which revealed a compact globular particle in solution (see Supplementary Figures S2, S3; Supplementary Table S1).

### Atomic absorption spectroscopy analysis

In 2003, one of the first studies regarding LanC’s (SpaC and NisC) revealed one bound zinc ion per protein (Okeley et al., 2003). Binding of this zinc ion is essential for the activity as verified by mutational studies (Li and van der Donk, 2007; Hegemann and Roderich, 2021). Therefore, we also investigated purified His_6_-MadC regarding the amount of zinc ions in solution *via* atomic absorption spectroscopy (AAS). We determined a zinc amount of 0.108 mg/L per 0.1 mg/ml of MadC resulting in a stoichiometry of 1:1 (see Supplementary Figure S4A). Since we used cobalt ions in the chelating chromatography, we also investigated its potential presence by AAS. No cobalt ions were detected in the purified MadC sample (see Supplementary Figure S4B).

### Crystal structure of MadC and a comparison with the AlphaFold2 models of other class I LanC’s

For crystallization, the heterologously expressed protein was freshly purified. During SEC the buffer was changed to HEPES buffer (20 mM HEPES, pH 8). Crystals were obtained using a 12 mg/ml His_6_-MadC protein solution and diffracted to a resolution of 1.7 Å. For phasing, the high-resolution dataset of MadC and the protein sequence were used as input files for the Auto-Rickshaw web server using molecular replacement mode.^1^ Afterwards, the initial model was manually further built and refined using COOT and subsequent refinement was performed using REFMAC 5^2^ and PHENIX.^3^ The final MadC structure was refined to 1.7 Å and the resulting R_work_ and R_free_ values were 19.0 and 22.4% (see Supplementary Table S2 for data statistics).

**Figure 2:**
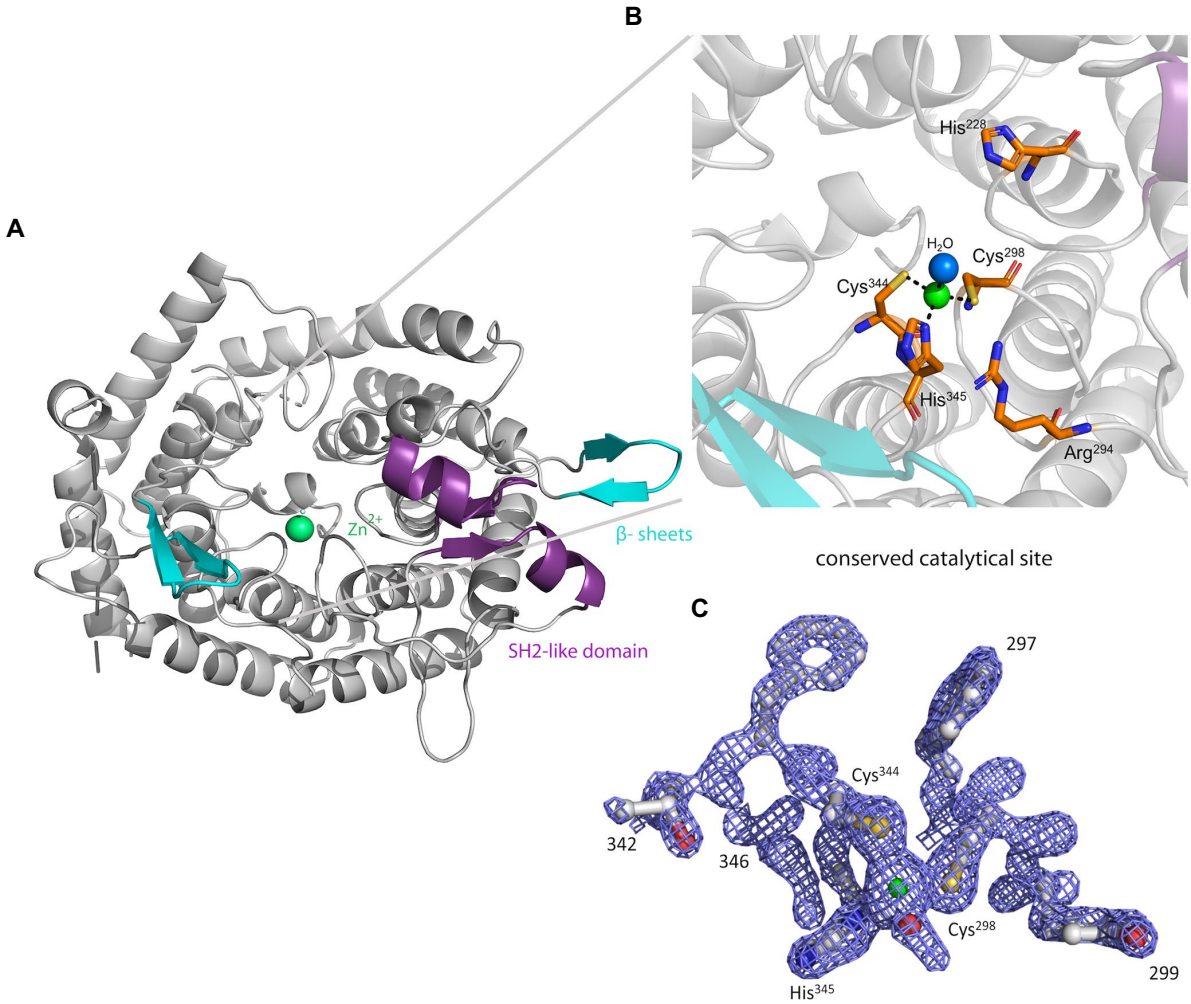
The crystal structure of MadC from *Clostridium maddingley*. **(A)** The crystal structure of MadC was determined at 1.7 Å. View down the spindle axis of the toroid structure colored in light-gray. The SH2-like domain is colored in magenta and the extending β-sheets are colored in cyan. The green-colored catalytic zinc ion (Zn^2+^) lies central inside the α,α-toroid structure and is adjacent to a β-sheet and the SH2-like domain. **(B)** Close-up view of the catalytic site with the toroid in light-gray. Conserved amino acids in the catalytic site are colored in elemental color-coding. Catalytic zinc ion is displayed in green and the coordinated water molecule in light blue. **(C)** Omit map (contoured at 2 σ) of residues at catalytical site and zinc ion. Zinc ion is displayed in green. The residues are colored in elemental color-coding. The red sphere beside the zinc ion is a oxygen atom from the water molecule. Cartoons were generated in PyMOL (www.pymol.org).

**Figure 3:**
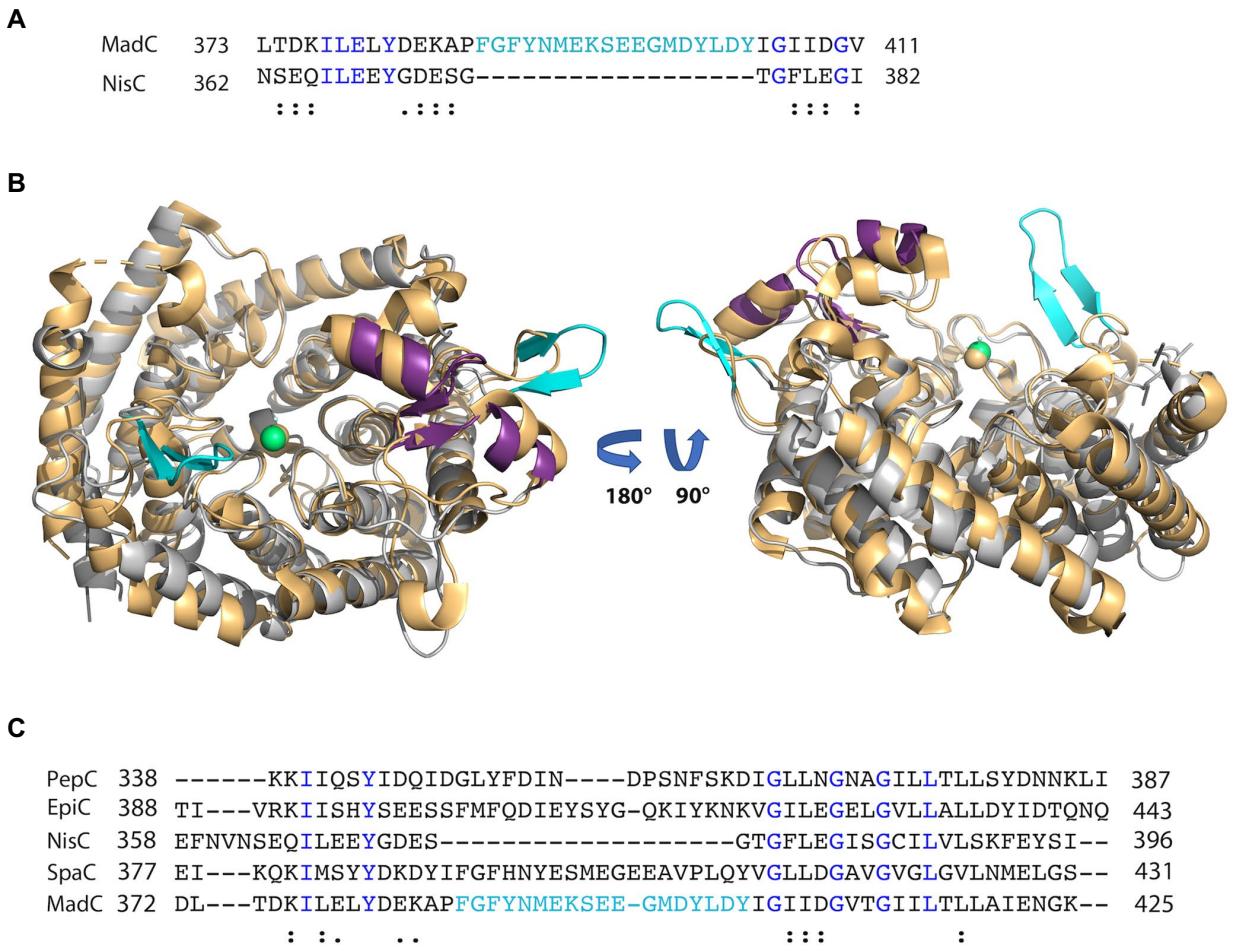
Alignment of class I LanC’s and structural comparison. **(A)** Sequence alignment of MadC and NisC *via* Clustal Omega. Blue colored amino acids indicate identical amino acids for this 2 sequence alignment, “:” indicates very similar amino acids; “.” indicates similar amino acids. Cyan colored amino acids display the β-sheet element near the catalytical zinc ion in MadC. **(B)** Structural superimposition of NisC (golden; PDB: 2G02) and MadC. MadC is colored in light grey except of the SH2-like domain (magenta) and the extended β-sheets in cyan. The alignment of the structures was performed in PyMOL. **(C)** Sequence alignment of selected class I LanC’s *via* Clustal Omega. Blue colored amino acids indicate identical amino acids for this specific alignment, “:” indicates very similar amino acids; “.” indicates similar amino acids. Cyan-colored amino acids display the β-sheet element near the catalytical zinc ion in MadC.

The crystal structure of MadC from *C. maddingley* revealed an α,α-toroid structure consisting of seven helices each (in total 14 helices), a catalytical zinc ion and a SH2-like domain as already described for NisC (see Figure 2; Li et al., 2006). The SH2-like domain is assumed to bind the leader region of maddinglicin and has high resemblance to eukaryotic peptide-binding domains (Bradshaw and Waksman, 2002). Unfortunately, the amino acids from position 26 to 45 could not be modelled due to poor electron density in this region. Interestingly, the NisC structure also revealed poor electron density (aa position 28 to 32) in the same region, which leads to the assumption that this region represents a highly flexible part in LanC’s. Additionally, the N-terminal thrombin cleavage site was also not observed in the electron density. The zinc ion is coordinated by two cysteines (Cys^298^; Cys^344^), one histidine (His^345^) and one water molecule (Figures 2B, C). Beside the conservation of the coordinating residues also the histidine (His^228^) and arginine (Arg^294^) residues, which are proposed to be essential for the acid/base chemistry are conserved (Figure 2B). An alignment of the tertiary structure of MadC and NisC (PDB 2G02) resulted in high similarity with a root mean square deviation (RMSD) of 1.2 Å for 254 Cα atoms (see Figure 3B). Further structural alignments showed resemblance to the crystal structure of the human LanCL1 (RMSD: 4.6 Å for 250 Cα atom pairs; PDB: 3E6U), which is a human homolog of LanC and also revealed resemblance to NisC (Zhang et al., 2009).

Interestingly, an extended β-sheet (aa 391–402) is present at the opposite site of the SH2-like domain, which was so far not observed in class I LanCs (see Figure 2A, highlighted in cyan). The zinc ion is located in the center between these two structural features. Furthermore, a sequence alignment of NisC and MadC highlights the absence of especially this structural feature in NisC (see Figures 3A, B) while highly similar amino acids frame the sequence in the alignment. Alignments with additional known class I LanC revealed neither a conserved lack of this domain nor a conservation of many amino acids of this particular structural feature in all these LanC’s (see Figure 3C).

**Figure 4:**
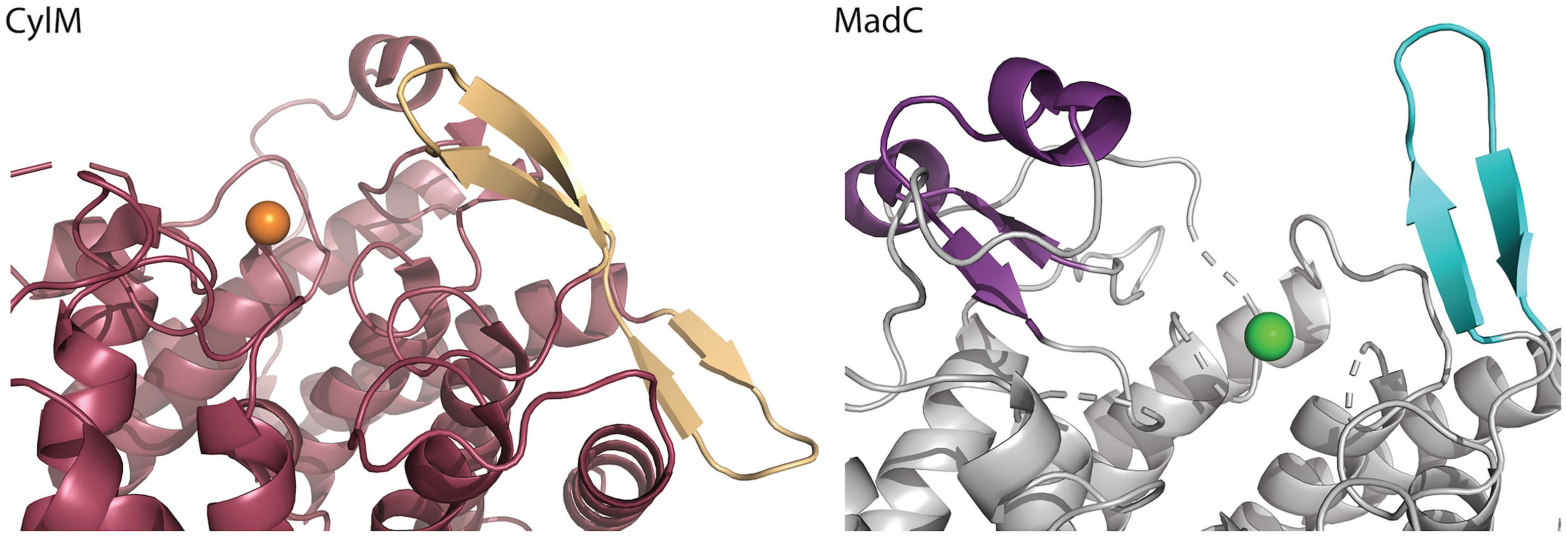
Comparison of the β-sheet domain of class I LanC’s and class II cyclization domain. In the zoom-in of the cyclase domain of CylM (residues 624–992 of CylM, PDB: 5DZT) the structure is colored in dark red except of the β-sheet domain (colored beige) close to the zinc ion (orange sphere). The α,α-toroid structure of MadC (PDB:8BYK) is shown in grey and the β-sheet close to the zinc ion (green sphere) is highlighted in cyan. The SH2-like domain is highlighted in purple.

A similar β-sheet could be observed for the class II CylM enzyme, which compared to class I cyclases lacks the SH2-like domain (see Figure 4; Dong et al., 2015). It is proposed that the β-sheet region of CylM is a putative binding site of the leader sequence due to the resemblance of anti-parallel β-sheet elements, for example, of class I NisB (Ortega et al., 2015). On the contrary, mutational studies of the class II LanM enzyme HalM2 highlight the independency of the dehydration domain regarding activity due to the absence of the cyclase domain, which also includes the putative similar anti-parallel β-sheet element as found for CylM (Rahman et al., 2020). Based on these results, it was assumed that the β-sheet element could not be responsible for substrate binding as previously assumed for CylM. However, independent activities for separated domains of a LanM enzymes [BovM (Ma et al., 2015)] and the impact on the K_D_ value whether the cyclase domain is present (e.g., K_D_: 6.0 ± 0.6 μM) or absent (e.g., K_D_: 47.8 ± 5.7 μM; Rahman et al., 2020) imply that a role in substrate binding still cannot be excluded. In comparison to CylM, MadC does not lack the SH2-like domain, which would lead to the assumption that no further binding domain(s) are essential for substrate binding. However, the anti-parallel β-sheet could still be relevant for substrate binding, specificity or coordination due to its close proximity to the zinc ion and due to the fact that until now the proposed function of the SH2-like domain was not confirmed experimentally. If the SH2-like domain is essential for substrate binding, the β-sheet can still influence the specificity of MadC or could be essential for interaction with the corresponding dehydratase.

**Figure 5:**
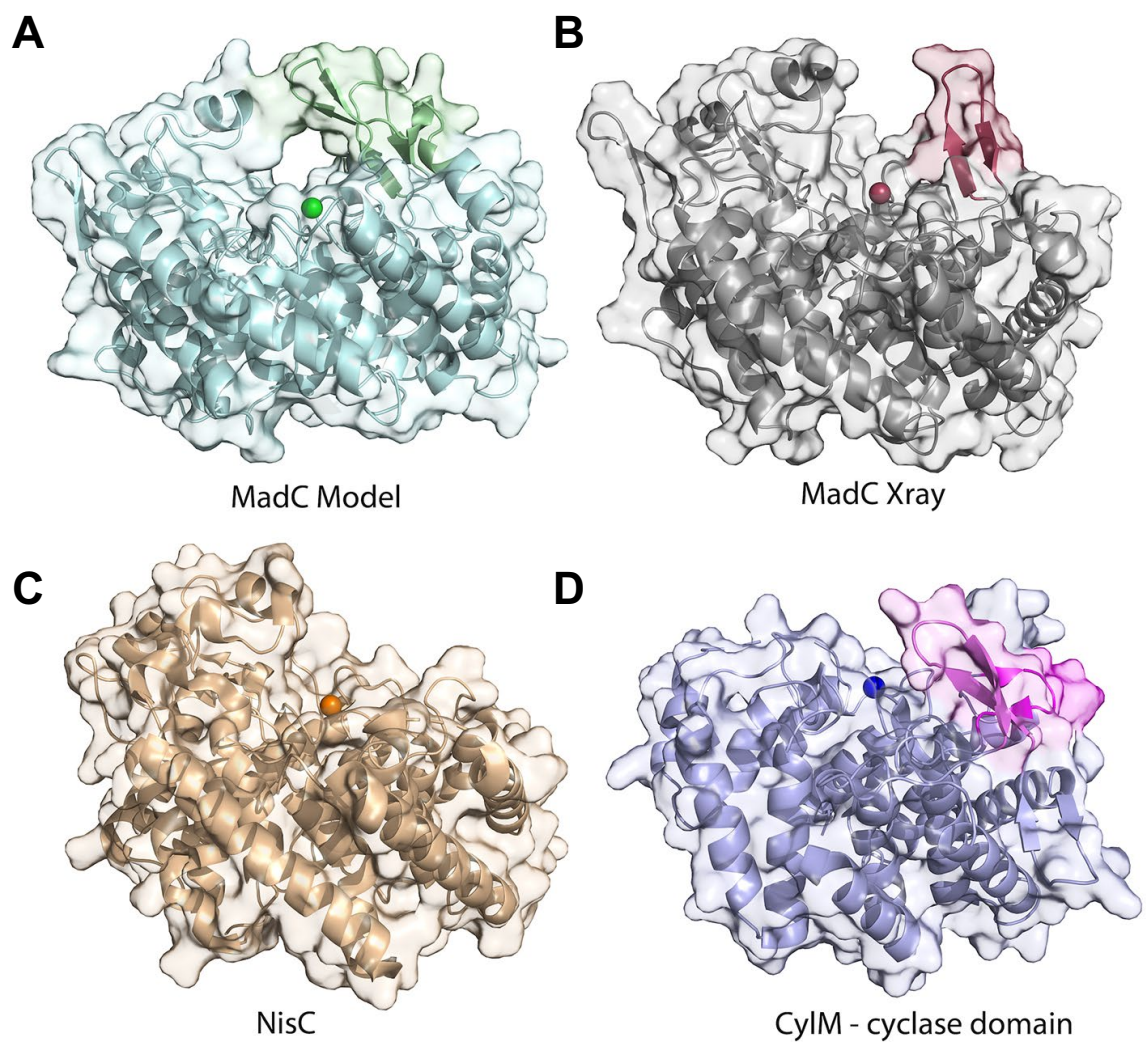
Comparison of structures of class I cyclases and a cyclization domain. **(A)** The AlphaFold2-model of the class I cyclase MadC. **(B)** The crystal structure of MadC was solved to a resolution of 1.7 Å. (PDB:8BYK). **(C)** The crystal structure of the class I cyclase NisC from the nisin system (PDB: 2G02). **(D)** The crystal structure of the cyclase domain of the class II modification enzyme CylM (PDB: 5DZT).

**Figure 6:**
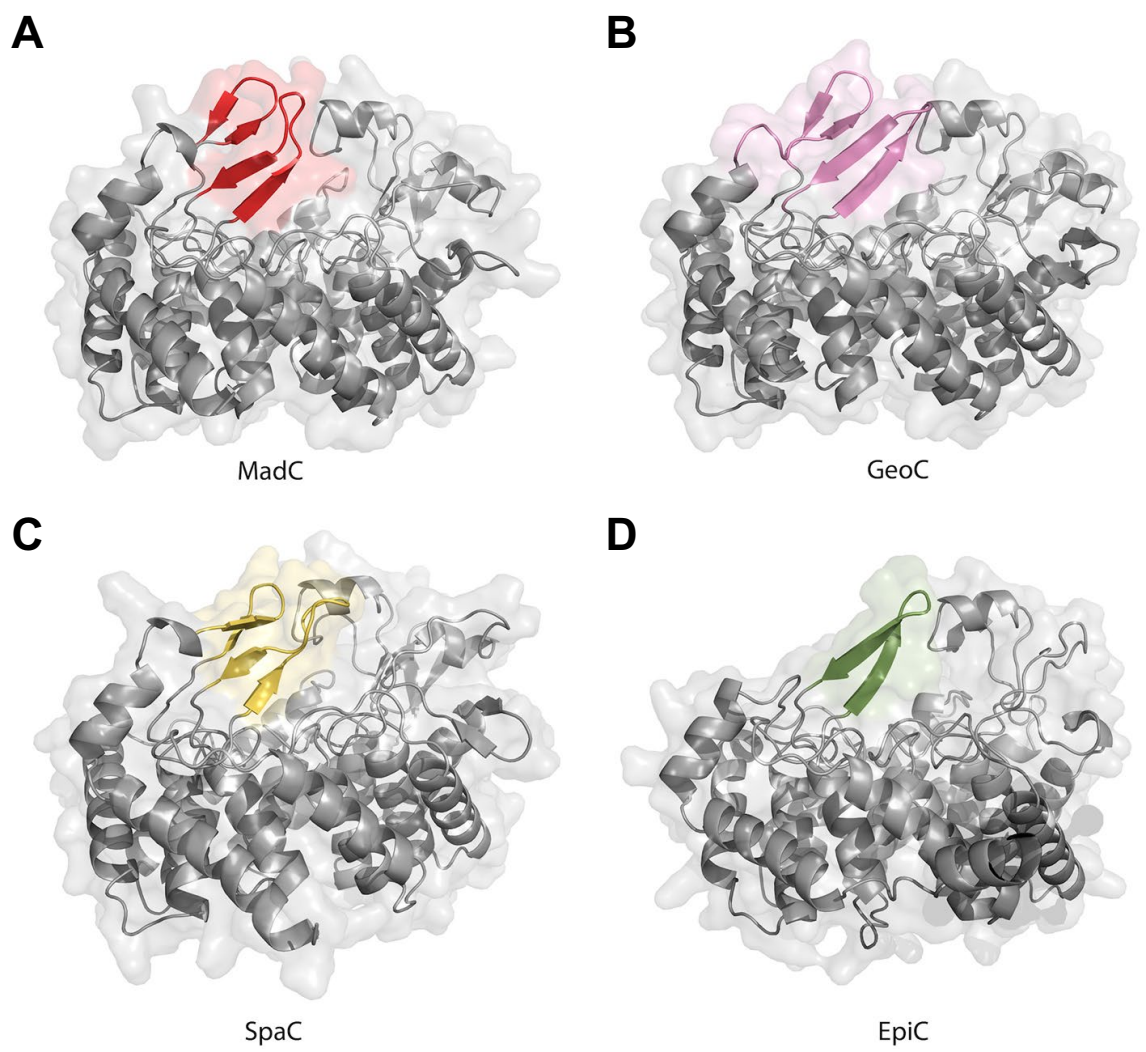
AlphaFold2 models of class I cyclases. **(A)** Class I cyclase MadC from Clostridium maddingley. The new features are colored in red. **(B)** Class I cyclase GeoC from Geobacillus thermodenitrificans. The new predicted features are in pink. **(C)** Class I cyclase SpaC from Bacillus subtilis. The new features are colored in yellow. **(D)** Class I cyclase EpiC from Staphylococcus epidermidis. The new feature is colored in green.

The lack of 20 amino acids in the X-ray structure of MadC (aa 26–45), most of which are also not present in NisC (data not shown, alignment in Supplementary Figure S5), led to the creation of an AlphaFold2 model of MadC (Jumper et al., 2021; Mirdita et al., 2022). A RMSD of 0.4 Å for 368 Cα atoms was determined between the model and the obtained crystal structure, which highlights the quality of the model. The quality or confidence of the model can also be valued with the parameter plDDT (range 0–100). The entire structure has mostly values between 80 and 100, has no value lower as 60 except of the N-and C-termini (see Supplementary Figure S6). In the previously unknown areas (residues 26–45 and residues 391–402) the value is around 80, which is in the range of 70–90, in which a generally good backbone prediction is expected. In this model, the missing aa 26–45 are predicted to form an additional anti-parallel β-sheet element, which is closer to the SH2-like domain than the previously mentioned anti-parallel β-sheet domain (see Figure 5B colored in dark red) and seems to form a tunnel-like surface above the zinc ion in combination with the SH2-like domain (see Figure 5A colored in green). The missing electron density for these amino acids could be explained by a high flexibility in the ligand-free (apo state) which could be shifted to a more ordered conformation after binding of a substrate. In addition, this anti-parallel β-sheet could also be involved in substrate binding or specificity. To compare LanC’s probably similar to MadC, BAGEL4 and RiPPMiner was used to search for lantibiotics similar in structure or sequence (Agrawal et al., 2017, 2021; van Heel et al., 2018). Geobacillin I was the lantibiotic with the highest sequence similarity and therefore the corresponding cyclase GeoC was predicted by AlphaFold2 (Jumper et al., 2021; Mirdita et al., 2022). The similarity can also be seen in the models, which share similar β-sheet features near the predicted catalytical site and manifest RMSD of 0.47 Å for 378 Cα atoms [see Figure 6A (red colored), B (pink colored)]. In this model, the plDDT value was for the β-sheet features lower than for the MadC model (around 60–80). All plDDTs between 50 and 70 should be treated with caution because low confidence is predicted (see Supplementary Figure S7). The feature at the C-terminus (residues 388–400) has a higher tendency to lower plDDT values than the feature at the N-terminus (residues 28–41). To evaluate whether these features are unique or were just not known due to the lack of available structures, AlphaFold2 models of two other class I cyclases (SpaC, EpiC) were generated (see Figures 6C, D). Surprisingly, the model of SpaC also contained both β-sheet domains even though the structural composition of subtilin is more similar to nisin (5 rings and hinge region) than to the predicted structure of maddinglicin [7 rings and no hinge region; see Figure 6C (yellow colored)]. Here, the structural feature at the N-terminus has higher plDDT values (range 70–80 for residues 37–45) than the C-terminal feature (range 60–70 for residues 396–408; see Supplementary Figure S8). In contrast, the EpiC model showed only one β-sheet feature (RMSD of 1.46 Å for 288 Cα atoms; see Figure 6D), which is predicted to be modelled with high accuracy (plDDT >80 for residues 407–418, see Supplementary Figure S9). Therefore, other areas seem to have lower confidence (e.g., N-terminus), which are not of interest in this study.

**Figure 7:**
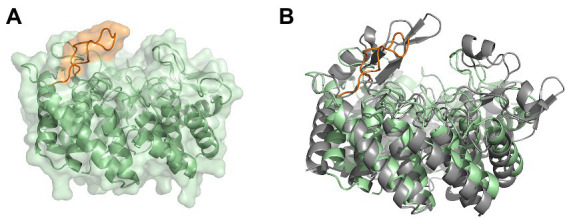
The modeled cyclase domain (residues 549–899) of SgbL from *Streptomyces globisporus* sp. NRRL B-2293. **(A)** AlphaFold2 model of the cyclase domain of the class IV modification enzyme SgbL. **(B)** Superimposition of the MadC crystal structure and the AlphaFold2 model of the cyclase domain of SgbL. MadC is colored in light grey.

Furthermore, we modelled a cyclase domain of a class IV modification enzyme (SgbL), which also contains the conserved amino acids for binding a zinc ion in contrast to class III modification enzymes (see Figure 7A). Even though the determined RMSD of the cyclase domain to MadC was quite high (5.24 Å for 161 Cα atoms), the model revealed also 14 helices forming an α,α-toroid structure. Interestingly, no SH2-like domain was present, but a protruding loop in the similar location as for the β-sheet element (residues 391–402) in MadC was observed. The protruding loop carries for some amino acids the lowest plDDT values (between 40 and 90) in the entire AlphaFold2 model of SgbL except of the C-terminus (see Supplementary Figure S10). In contrast, the remaining parts of SgbL shows plDDT values between 80 and 100, which represents a good backbone prediction as mentioned before. In this case, the secondary structure of the loop in SgbL (colored orange in Figure 7A) is still under discussion. However, the location of these amino acids within SgbL is based on the higher reliability of the remaining amino acids more likely to be reliable.

In summary, no correlation between substrate and the occurrence or lack of the new discovered β-sheet features of the corresponding cyclases exists.

**Figure 8:**
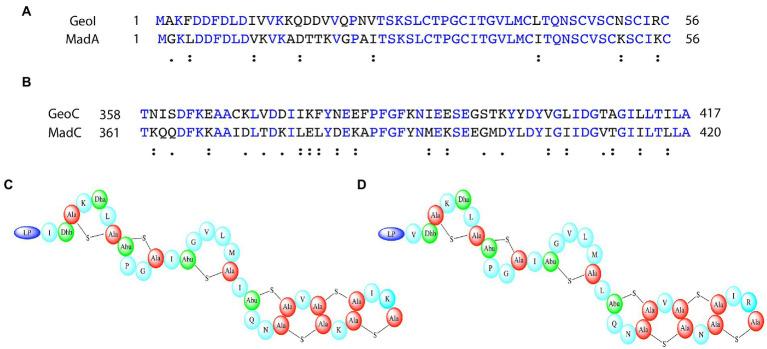
Comparison of maddinglicin and geobacillin-I and the corresponding cyclases. **(A)** Sequence alignment of MadA and GeoI *via* Clustal Omega. Blue colored amino acids indicate identical amino acids for this 2 sequence alignment, “:” indicates very similar amino acids; “.” indicates similar amino acids **(B)** Sequence alignment of MadC and GeoC *via* Clustal Omega. Only the part including the β-sheet domain of MadC is displayed for comparison. Blue colored amino acids indicate identical amino acids for this 2 sequence alignment, “:” indicates very similar amino acids; “.” indicates similar amino acids **(C)** Proposed structure of MadA. **(D)** Solved structure of GeoI (Garg et al., 2012).

Alternatively, the β-sheet could be essential for the predicted 4 intertwined rings at the C-terminus of MadA. To predict the rings in maddinglicin RiPPMiner was used (Agrawal et al., 2017, 2021). Furthermore, BAGEL4 and RiPPMiner also identified structurally related or sequence similar lantibiotics such as geobacillin I (GeoI; van Heel et al., 2018). Interestingly, MadA and GeoI share a high degree of sequence identity (73.2%) and similarity (87.5%; see Figure 8A). Due to the high similarity, these lantibiotics likely share the same ring architecture (or also similar ring size), which was already verified for GeoI *via* tandem MS and NMR studies (see Figure 8D; Garg et al., 2012). An alignment of MadC and GeoC revealed high conservation of the β-sheet sequence of MadC, which implies that the structural feature could form the basis for the observed ring pattern of these lantibiotics (see Figure 8B). Further studies are required in the presence of a cyclase mutant lacking the β-domain to verify the importance of this subdomain for the formation of these four intertwined rings. These four intertwined rings at the C-terminus are likely the molecular reason for the higher stability of GeoI compared to NisA (Garg et al., 2012), which could contain high potential for future synthetical design of lantibiotics.

### MadC:substrate interactions measured by isothermal titration calorimetry

Isothermal titration calorimetry (ITC) was used to identify the importance of amino acids of the LP or of the CP for substrate binding to LanC enzymes using MadC as an example. In previous studies of NisC, the importance of the FxLx-box inside the LP independent of the modification state of the lantibiotic was demonstrated *via* ITC (Abts et al., 2013). It was suggested that the modification state of the pre-LanA has an influence on the affinity and binding to LanB [for example NisB (Mavaro et al., 2011)] and the LanBCA complex [for example NisBCA from *L. lactis* (Reiners et al., 2017)]. In this study, the dependence of the CP was examined using the previously mentioned leader constructs of MadA.

**Figure 9:**
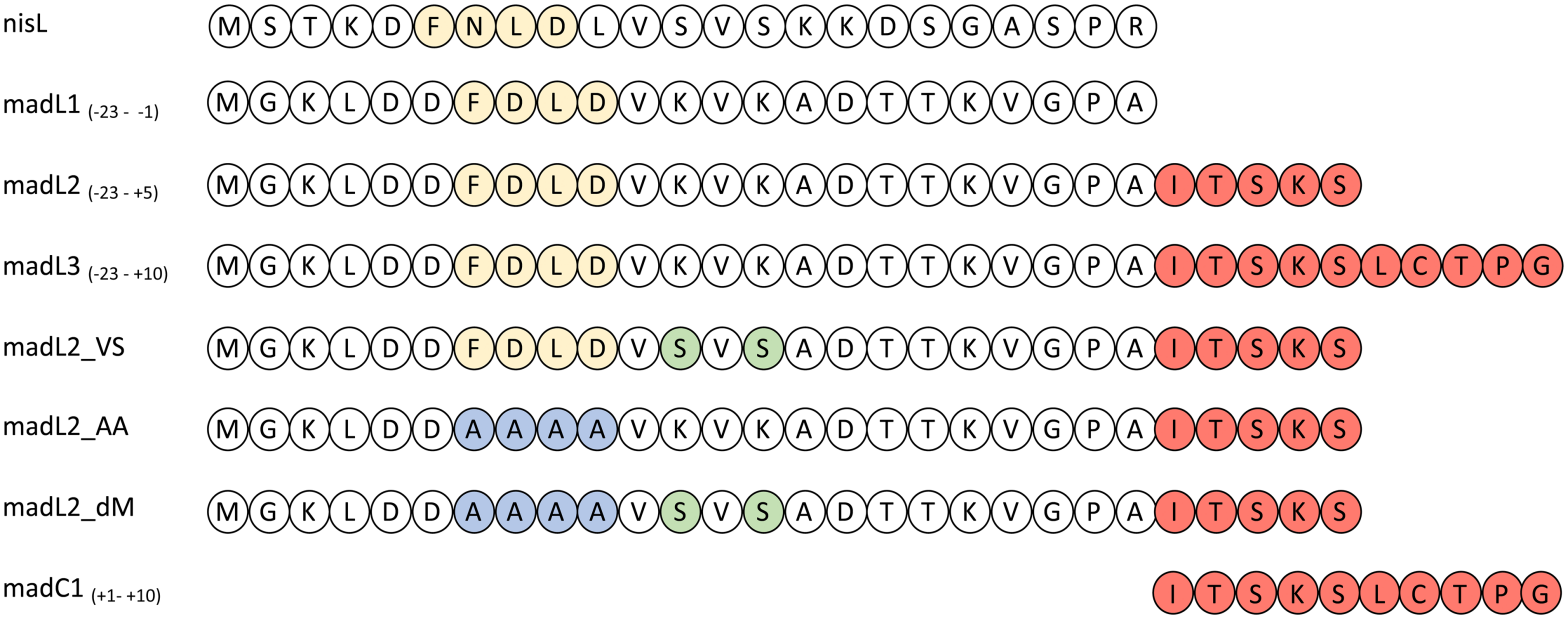
Overview of nisin LP (nisL) and maddinglicin leader variants (madL1-3, madC1, madL2_VS, madL2_AA, madL2_dM). The recognition box (-FxLD-) of the PTM enzymes is colored in light yellow. The amino acids from the CP are colored in light red. The one-letter code for amino acids was used. In the constructs the mutation of the FDLD-motif into-AAAA-is highlighted in blue and the mutations from lysine to serine are highlighted in light green.

The importance of the leader or more precisely the FxLx-box inside the LP sequence for substrate binding has been clearly demonstrated (Abts et al., 2013; Thibodeaux et al., 2015). Therefore, synthesized LP constructs of maddinglicin named as L1, L2, L3 and mutants (L2VS, L2AA, and L2dM) were designed (see Figure 9).

The LP-only (madL1) revealed a binding affinity of 10.5 ± 4.3 μM (see Table 1, see Supplementary Figure S11A). In comparison, the affinity of NisC to its cognate LP (3.8 ± 0.6 μM) was significantly higher (Abts et al., 2013). The longest construct (madL3), which also contains the first cysteine of the putative ring A, revealed the highest affinity with 5.7 ± 1.7 μM, which is nearly twice as high as the affinity of NisC to the unmodified precursor peptide nisA (2.4 ± 0.5 μM; see Table 1; Abts et al., 2013). Nevertheless, MadC revealed affinities in the same concentration range as NisC. The stoichiometry for the LP L1, L2, L3 and the mutant L2VS were determined to 1:1. The affinities of madL2 to MadC (9.35 μM ± 3.92 μM) and madL1 to MadC (10.50 μM ± 4.26 μM) indicated no influence of the first five CP amino acids (see Table 1). MadL3 has a lower average K_D_ value compared to the other LP constructs, which leads to the assumption that the addition of the first cysteine in the CP has a slight effect on the binding affinity. However, madL1 and madL2 also have higher standard deviations.

**Table 1:** ITC data of MadC and the LP variants.

Peptide constructs	N	K_D_	Δ H	-TΔ S	Δ G
		[μM]	[kJ/mol]	[kJ/mol]	[kJ/mol]
Leader 1_(−23 – −1)_	0.83 ± 0.06	10.4 ± 4.26	−36.67 ± 2.08	13.04 ± 11.9	−30.12 ± 2.89
Leader 2 _(−23 − +5)_	0.99 ± 0.09	9.35 ± 3.92	−25.20 ± 6.56	11.53 ± 9.72	−27.70 ± 0.92
Leader 3 _(−23 − +10)_	0.99 ± 0.04	5.69 ± 1.72	−30.55 ± 3.27	13.24 ± 4.46	−31.96 ± 3.43
Leader 2 VSVS	0.99 ± 0.05	8.20 ± 2.50	−56.93 ± 1.22	20.26 ± 5.49	−36.66 ± 1.07
Leader 2 AAAA	No binding observed in ITC measurements				
Leader 2 dM	No binding observed in ITC Measurements				
Core peptide _(+1 − +10)_	No binding observed in ITC Measurements				
Nisin leader	No binding observed in ITC measurements				

All experiments were performed at least in triplicate, and the error represents the standard deviation of a minimum of three independent experiments. For experimental details, see Materials and Methods.

**Figure 10:**
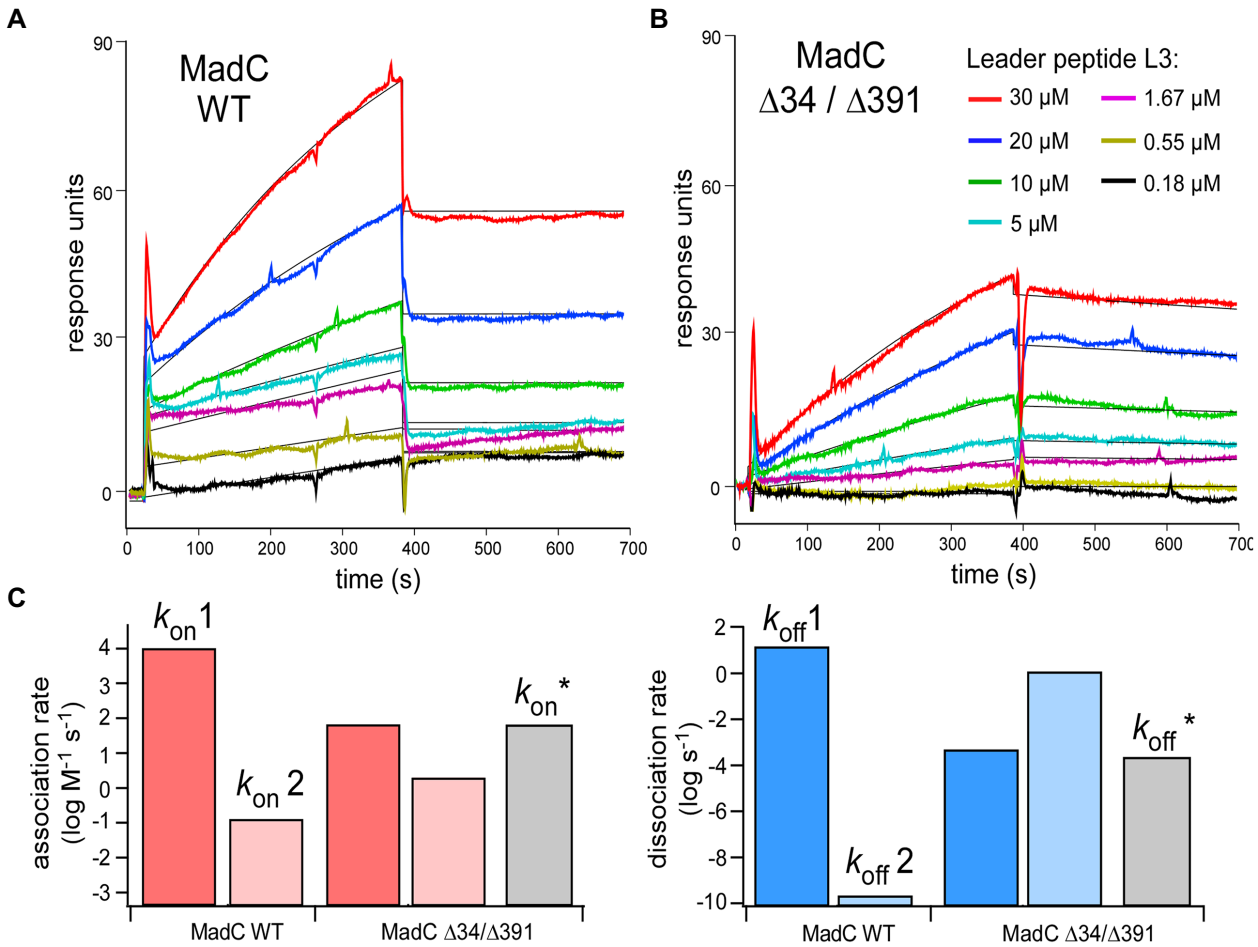
**(A,B)** SPR sensorgrams of MadC:L3 binding recorded for the wild-type and ∆34/∆391 mutant. The color coding for L3 concentrations is provided in the panel **(B)** Black lines: Fitting to the kinetic model (1:1 binding, two states). **(C)** Association (left, red) and dissociation (right, blue) rates acquired from the fitting. Grey bars: Association/dissociation rates for MadC ∆34/∆391 mutant acquired from simple 1:1 binding model.

Comparison of the LP sequence of nisin and maddinglicin identified an interesting VxVx-pattern upstream of the FxLx-motif. The “x” in the unique pattern corresponds to the amino acid serine (S) in the case of nisin and to lysine (K) in case of maddinglicin. The difference in size and in charge of these amino acids led to the creation of the mutant madL2_VS in which the VKVK-motif of maddinglicin was mutated to the corresponding VSVS-motif of nisin (see Figure 9). Surprisingly, the madL2_VS mutant revealed a similar stoichiometry and affinity as madL2 (see Table 1; Figures 10B, D). Contrary results were observed for the mutants madL2_AA, in which the FxLx-motif was mutated to four alanines, and madL2_dM, which carries the mutations of madL2_VS and madL2_AA (see Figure 9). The mutation of the FDLD-motif into four alanines (madL2_AA) led to a complete loss of binding (see Figure 10F). Due to the fact that the double mutant also carries this mutation, the ITC measurements demonstrated again no binding. Additionally, the lack of the LP by using only 10 aa of the CP (C1) showed no binding even though it contains the cysteine of the first putative ring compared to MadL2_AA, which could lead to binding at the zinc ion (see Figure 10E). Therefore, we conclude that the FxLx-motif seems to be crucial for substrate binding of MadC. Even though the mutant MadL2_VS revealed no difference in stoichiometry and affinity to MadC, the measurement with the nisin LP demonstrated no binding at all. In conclusion, the LP of MadA possesses a potential important amino acid or region beside the FxLx-motif, which has a severe impact on the substrate selectivity of MadC. It also needs to be considered that one leucin is positioned between the FxLx motif and the VSVS motif in nisin and contrary the VKVK motif of maddinglicin follows right after the FxLx motif. The shift of this motif could have a higher impact on the binding by MadC than the simple mutation of single amino acids.

### MadC:substrate interactions by surface plasmon resonance

To investigate the relevance of the β-sheets (residues 26–45 and residues 391–402) in substrate binding or specificity of MadC, the single mutants MadC ∆34 (residues 34–41 deleted, inserted GAG-linker), MadC ∆391 (residues 391–402 deleted, inserted GAG-linker) and the double mutant MadC ∆34 ∆391 (carrying both mutations) were created (see Material and Methods). Due to lower yield of purified MadC mutants in comparison to the wildtype MadC (purification in Supplementary Figure S12), we performed SEC-MALS analysis. Surprisingly, binding for all MadC variants (data not shown) similar to wildtype was observed, which suggested no influence on the ability of substrate binding itself. Nevertheless, the SEC-MALS method is limited to end-point determination of substrate binding incapable on determining affinities or kinetic values. While a direct functional *in vitro* assay is lacking for MadC and the yield of purified MadC mutants were not sufficient for ITC analysis, we employed surface plasmon resonance (SPR) to characterize the affinity and the kinetics of the leader peptide binding to MadC. For this we used madL3 and the wild-type MadC protein. The protein was anchored on the sensor surface *via* Ni-NTA:His-tag coupling, and madL3 was injected at concentrations ranging from 185 nM to 30 μM. The MadC:madL3 interaction was clearly resolved, showing both the association and dissociation phases on the sensorgrams (Figure 10A). Based on the maximum RU values, an affinity of 16 ± 7 μM was determined. The affinity was reduced in comparison to the ITC measurement above, which could be due to the immobilization of MadC on the SPR sensor surface. Next, we analyzed the LP binding to the MadC variant ∆34/∆391 where both β-sheets elucidated in the comparison of crystal structure and AlphaFold2 model of MadC were deleted (residues 34–45 and residues 391–402; Figure 10B). For the mutant, 2.5-fold lower *K*_D_ values with 35 ± 7 μM was determined. While the change in the affinity was modest, the association/dissociation kinetics for the two MadC variants was clearly different. A clear two-phase behavior was observed for the wild-type MadC, as interactions with the LP initially caused a rapid response followed by a slower saturation phase. Differently, sensorgrams for the MadC ∆34/∆391 variant showed only the slow phase. To account for the complex binding mode of the wild-type MadC, all sensorgrams were fitted by 1:1 binding model assuming two states/conformation. As expected, two different binding modes were resolved for the wild-type cyclase, as the association rates differed by five orders of magnitude (Figure 10C). The values determined for the mutant were similar in between, and matched closely the association rate derived from the simple 1:1 binding model. Thus, we concluded that the feature b-sheet domains must contribute to recognition and binding of the substrate.

## Discussion

Lanthipeptides, one class among RiPPs, contain (methyl-)lanthionine rings, which can be installed either by a single protein (LanM, LanL, LanCK), two proteins (LanC and LanB) or three proteins (LanK, LanX, LanY). Here, we determined the structure of another class I lantibiotic cyclase (MadC) with structural elements (anti-parallel β-sheets) of unknown functionality, which are also found among other class I cyclases based on their structural models obtained by AlphaFold2. A protruding loop of a class IV cyclase domain model in a similar area of one of the β-sheet elements in MadC could also be discovered, which raises the question how important these anti-parallel β-sheet elements are for substrate specificity or ring formation, respectively. Before this study, the crystal structure of NisC was the only representative structure of bacterial class I cyclase. However, our results highlight that NisC seems to be unique among class I cyclases. The comparison with other class I cyclases (EpiC, SpaC, GeoC and MadC) emphasizes that NisC so far is the only cyclase lacking both β-sheet elements. Still, the partly lower confidence in the features of some AlphaFold2 models needs to be considered. However, the existence of more amino acids in the sequence and the higher accuracy for the toroid structure cannot be denied, which concludes that the amino acids are present in this area although the secondary structure is still under discussion.

Even though subtilin and nisin share a high structural similarity, the AlphaFold2 model of the corresponding cyclase SpaC possesses both anti-parallel β-sheets near the active site. The lantibiotic epidermin is due to the S-(2-aminovinyl)-D-cysteine at the C-terminus the most structural distinct lanthipeptide in this comparison and the corresponding cyclase just lacks one anti-parallel β-sheet, which is in closer proximity to the SH2-like domain than the other. One can assume that the absence of specially this β-sheet correlates with the special modification (S-(2-aminovinyl)-D-cysteine), which installation probably requires more space than smaller modifications such as (methyl-)lanthionine rings (see Supplementary Figure S13). The high similarity of the MadC and GeoC model is not surprising, as both the sequences and predicted lantibiotic structures are highly similar. Based on the known or predicted structures of the lantibiotics maddinglicin, geobacillin I, and epidermin, which share their last rings at the extreme C-terminus, we propose that the occurrence of the β-sheet elements in the corresponding cyclases could be related to occurrence of the intertwined ring formation at the C-terminus of lantibiotics. However, SpaC also contains these structural features and the corresponding lantibiotic, subtilin, resembles more the structure of nisin. Therefore, no connection between the ring pattern at the C-terminus of the lanthipeptide structure and the existence of the β-sheets can be made. Nevertheless, one can hypothesize that they are relevant for substrate specificity and ring size limitation or specification, respectively. The discovery that NisC can even cyclize non-lantibiotic peptides fused to the LP of nisin and the consequently promiscuity paved the way for the possibility to produce novel lantibiotics without the difficulties to obtain them from their natural source (such as fastidious or pathogenic strains) and evaluate for example their antibacterial activity against multi-resistant pathogens (Rink et al., 2007; Majchrzykiewicz et al., 2010; van Heel et al., 2016). The lack of the β-sheet elements of MadC in NisC could be one of the reasons why NisC has such a low substrate specificity. Additionally, NisC was so far not investigated regarding ring size limitation, and the knowledge about its uniqueness could then even pave the way for engineering more diverse lantibiotics, which can be still modified by the nisin modification machinery.

Furthermore, the SH2-like domain, predicted to bind the lanthipeptide, found in all class I cyclases is not present in class II or class IV cyclization domains and no evidence exist for its particular role in substrate binding. Because of the lack of the SH2-like domain in modification enzymes, which contain all modification functions in one enzyme (class II, class IV) and the conserved amino acids in the cyclisation domains of class I cyclases, one could speculate that the SH2-like domain is relevant for complex formation with LanB’s instead of substrate binding. Complex formation is not necessary for the modification enzymes LanB or LanC to be active, but *in vivo* and *in vitro* studies highlight the presence and relevance of this complex formation (Siegers et al., 1996; Kiesau et al., 1997; Xie et al., 2002; Reiners et al., 2017; Lagedroste et al., 2020). In more detail, kinetic studies of NisT from Lagedroste et al. investigated the impact of NisB and NisC on the secretion rate (Lagedroste et al., 2020). Here, the presence of the PTMs of nisin (NisB and NisC) increased the secretion rate significantly. However, the deletion of NisB and NisC only resulted in a lower secretion rate (4-fold lower compared to the strain with NisB and NisC), not in abolishment of secretion. Therefore, secretion still was possible without the PTMs, but with a significant impact on secretion efficiency.

In our study, ITC showed that the leader peptide of maddinglicin is necessary and the core peptide has minor influence on the binding affinity of MadC. Furthermore, it seems that specifically the “FxLx” motif is highly important since the madL2_AA mutant showed no binding at all, while the mutations in madL2_VSVS had no major effect on binding affinity. Surprisingly, the LP of nisin could not be recognized by MadC even though it shares similar motifs (FxLx, VxVx). The independence of the core peptide and the importance of the “FxLx” motif is in line with the observation of previous *in vitro* studies of the class I cyclase NisC and the class II modification enzyme HalM2 (Abts et al., 2013; Thibodeaux et al., 2015). However, HalM2 probably contains two binding sites, one within the dehydratase domain and one within the cyclase domain, which explains the influence of the maturation state of the substrate on binding affinity. This fact makes it difficult to compare the determined K_D_ values of a class II modification enzyme with the one’s of a class I cyclase. Therefore, further mutational studies and the structure of a cyclase in their substrate bound-state are required for more detailed insights.

The additional investigations of MadC wildtype and the β-sheet mutants (∆34, ∆391 and ∆34/∆391) *via* SEC-MALS and SPR revealed that the β-sheets do not play a major role in substrate binding. The deletion of the β-sheets did not result in “no binding” of the substrate as it was previously assumed. However, the SPR studies demonstrated that wildtype MadC has a two-state binding mode in contrast to the double mutant MadC ∆34 ∆391. These results lead to the assumption that the β-sheets could be more relevant for substrate recognition during the binding process and therefore contributes to substrate specificity of MadC. Additionally, due the lack of an *in vitro* assay for MadC the relevance of these features in ring formation or ring specificity cannot be excluded.

In conclusion, the class I cyclase MadC revealed high similarity to NisC regarding the oligomeric state in solution, substrate binding affinity and stoichiometry. However, the discovery of the previously unknown β-sheet elements, which could also be found in other class I cyclases, paved the way for further mutational studies regarding substrate specificity or ring pattern/modification limitations.

## Materials and methods

### Cloning of His_6_-MadC

For generating of a His_6_-MadC expression plasmid the Gibson assembly method was used (Gibson et al., 2009, 2010). Here a pET28b(+) vector was amplified *via* PCR with the primers FOR-pET28b(N.term Gibbs)XhoI and REV-pET28b(Nterm.Gibbs) to create a linear backbone product (see Supplementary Table S2). Beforehand, the sequence of MadC (NCBI: EKQ50560.1) was ordered in a pUC57 vector from GenScript (Netherlands). The amplification of the *madC* gene was performed with primers FOR-MadC(N.term) NdeI and REV-MadC(N.term) XhoI (see Supplementary Table S2). The plasmids containing the *madC* gene were sequenced and used for the following experiments after transformation in *E.coli* DH5α (Hanahan, 1983). The mutants were created with primers, which did not amplify the residues 34–41 (∆34) or residues 391–402 (∆391) and one of the primer pair carries an overhang sequence for the residues GAG as a linker (see Supplementary Table S3).

### Expression of MadC

The *madC* gene was cloned previously into a pET28b(+) vector resulting in a protein with an N-terminal His_6_-tag, containing a thrombin cleavage site. The resulting plasmid pET28b(+)_madC was transformed into *E. coli* BL21 (DE3) cells and plated on 2YT-Agar plates containing kanamycin (30 μg/ml). The agar plates were incubated overnight at 37°C. Precultures were prepared using 2YT-media (100 ml; 30 μg/ml kanamycin) with a cryo stock or freshly colonies from 2YT-agar plates. After overnight incubation, the main culture (1 l) was inoculated with the preculture to an OD_600_ of 0.1. The incubation was performed at 37°C and 180 rpm. Because of leaky expression no induction was required, yet after reaching an OD_600_ of 0.6–0.8 the main culture was incubated for the following 3–4 h. Afterwards, the cells were harvested at 5,000 g for 15 min at 4°C and subsequently frozen with liquid nitrogen for the temporary storage at-20°C.

The same procedure was used for the MadC mutants (MadC∆34, MadC∆391, MadC∆34∆391).

### Purification of MadC and MadC mutants

Cells were thawed at RT and resuspended in resuspension buffer [50 mM HEPES, 500 mM NaCl, 10% (v/v) glycerol, pH 8.0 (10°C)]. The cells were lysed using a M-110P cell disruptor (Microfluidics). Afterwards, cell debris were centrifuged at 4°C with a high spin step (60 min, 100,000 *g*) and the supernatant was taken. Imidazole was added to a final concentration of 20 mM.

#### Talon purification

The Talon purification was performed with a 5 ml HisTalon Superflow Cartridge (Takara), which was equilibrated with 10–50 ml IMAC low buffer [50 mM Hepes, 50 mM NaCl, 20 mM imidazole, pH 8.0 (10°C)] using an ÄKTA pure system (GE Healthcare). Subsequently, the cytoplasmic fraction after cell lysis and centrifugation was loaded on the column with a flow rate of 0.5–0.75 ml/min. After the loading step, the column was washed with 100 ml of IMAC low buffer at a flow rate of 1 ml/min. Hi6-MadC or the mutants were eluted by increasing the imidazole concentration from 20 mM to 150 mM in one step. A buffer exchange of the eluted His6-MadC variant with a pre-equilibrated PD10-column for storage was performed. For Otherwise, the eluted MadC variants were pooled and concentrated with a centrifugal filter unit (MW cut-off: 30 kDa) to a volume of 1 ml (≈15 mg/ml) for the following His_6_-tag-cleavage step *via* thrombin cleavage kit (Sigma Aldrich).

#### IMAC after thrombin cleavage

After an overnight incubation of the purified His_6_-MadC with the thrombin-resin according to the manual instructions, the sample was centrifuged at 500 × *g* for 5 min. The supernatant was collected in an Eppendorf tube. Afterwards, the resin was washed with low salt HEPES buffer and centrifuged again at 500 × *g* for 5 min. The supernatant was collected again. The washing step was performed twice. The collected supernatant was applied with a 5–10 ml loop onto a HisTalon Superflow Cartridge (Takara) column. The cleaved MadC eluted directly and was collected in the flow-through fractions, which were concentrated and further used for size exclusion chromatography. The non-cleaved His_6_-MadC still bound to the column and was eluted as described in “Talon purification.”

#### Size exclusion chromatography

The size-exclusion chromatography (SEC) of purified His6-MadC, His6-MadC mutants or MadC was performed with a pre-equilibrated Superdex 200 10/300 GL Increase *via* an ÄKTA pure system (GE Healthcare) at a flow rate of 0.5 ml/min. The buffer, which was used for the equilibration and elution step, was chosen according to the next step (e.g., ITC or SEC-MALS). The purity of the MadC sample was visualized and checked *via* SDS-PAGE (Laemmli, 1970; Towbin et al., 1979; Burnette, 1981).

### Isothermal titration calorimetry

To investigate the binding parameters between the cyclase MadC and leader variants Isothermal Titration Calorimetry (ITC) was used. The leader variants were ordered and produced by GenScript (Netherlands). To prevent heat resulting due to different buffer mixture, the enzyme and leader variants were separately dialyzed against the ITC /Dialysis buffer (50 mM HEPES, 500 mM NaCl, pH 7.5).

After dialysis, the concentration of the enzyme was determined *via* Nanodrop (Thermo Fisher) and adjusted to 200–600 μM of MadC. In contrast, the concentrations of the leader variants were determined *via* RP_18_-HPLC (Agilent Technologies) and afterwards adjusted to a concentration of 20–60 μM. All experiments were performed using an ITC200 instrument (Microcal, Malvern Panalytical). Before the measurement the maddinglicin leader variant was loaded with a volume of around 280 μl into the cell, whereas the enzyme MadC with a volume of 40 μl was used to fill the syringe.

All ITC experiments were performed at 25°C with 20 injections, in which 2 μl of MadC was pipetted from the stirring syringe (stirring speed = 750 rpm) into the cell containing the leader variant. The reference power during the measurement was 7 μcal s^−1^ and the spacing time between each injection was 150–180 s. Each experiment was performed at least in triplicate. The evaluation was done with the provided PEAQ software from the manufacturer (Malvern Panalyticals, Version 1.41).

### Surface plasmon resonance

SPR measurements were carried out on two-channel 2SPR system (SR7500DC, Reichert Inc.). MadC wild-type and mutants bearing hexa-histidine tags on their N-termini were immobilized on the surface of a poly-NTA derivatized sensor chip (NiHC1500M, XanTec Bioanalytics GmbH, Düsseldorf, Germany). Briefly, the surface of the chip was cleaned using 0.5 M EDTA (pH 8.5), then 5 mM NiCl_2_ in running buffer (200 mM NaCl, 50 mM HEPES/NaOH pH 8) was coupled to the surface, resulting in signal of 200 response units (RU). Individual MadC variants were injected in one channel at the flow-rate of 10 μl/min and captured on the surface to 400–500 RU. The second channel remained empty and served as a reference for unspecific interactions of the leader peptide with the surface. Serial dilutions of the leader peptide L3 from 30 μM to 180 nM were prepared in the running buffer and then injected for 6 min at the flow-rate of 25 μl/min followed by a dissociation phase of 10 min with the running buffer. At the end of each association/dissociation cycle the chip surface was cleaned by the injection of 0.5 M EDTA pH 8.5, followed by Ni2+ immobilization and MadC capturing. The cleaning/regeneration step was required due to the incomplete dissociation of the L3 peptide. For the wild-type and ∆34/∆391 mutant MadC the experiments were performed in biological duplicates. Kinetic analysis was performed using TraceDrawer 1.9 (Ridgeview Instruments AB, Uppsala, Sweden) and sensorgrams were fitted to a 1:1 two-state kinetic model. All sensorgrams were corrected by the subtraction of the reference channel and a buffer injection (blank).

### Small-angle X-ray-scattering

We collected SAXS data from MadC on a Xeuss 2.0 Q-Xoom system from Xenocs, equipped with a PILATUS 3 R 300 K detector (Dectris) and a GENIX 3D CU Ultra Low Divergence X-ray beam delivery system. The chosen sample to detector distance for the experiment was 0.55 m, results in an achievable q-range of 0.05–6 nm^−1^. The measurement was performed at 10°C with a protein concentration range of 4.4 to 13.8 mg/ml. The MadC samples were injected in the Low Noise Flow Cell (Xenocs) *via* an autosampler. We collected 12 frames with an exposer time of 10 min/frame and scaled the data to absolute intensity against water.

All used programs for data processing were part of the ATSAS Software package (Version 3.0.3; Manalastas-Cantos et al., 2021). Primary data reduction was performed with the program PRIMUS (Konarev et al., 2003). With the Guinier approximation (Guinier, 1939), we determine the forward scattering *I(0)* and the radius of gyration (*R_g_*). The program GNOM (Svergun, 1992) was used to estimate the maximum particle dimension (*D_max_*) with the pair-distribution function *p(r)*. Low resolution *ab initio* models were calculated with GASBOR (Svergun et al., 2001) with P1 symmetry. Superimposing of the MadC model was performed with the program SUPCOMB (Kozin and Svergun, 2001). The agreement with the MadC crystal structure was checked with the program CRYSOL (Svergun et al., 1995). We uploaded the SAXS data to the Small Angle Scattering Biological Data Bank (SASBDB) (Valentini et al., 2015; Kikhney et al., 2020), with the accession code SASDMT7.

### Multi angle light scattering

To determine the molecular weight and stoichiometry of the presumably cyclase MadC in solution a combination of size exclusion chromatography and multi-angle light scattering (SEC-MALS) was used. The analyses were performed on an Agilent 1260 HPLC System in combination with a triple-angle light scatter detector (miniDAWN TREOS) and a differential refractive index detector (Optilab rEX – both Wyatt Technology Europe).

Analysis of isolated MadC was performed by injection of 100 μl of a 20 μM solution. The sample was applied on a pre-equilibrated [MALS buffer (50 mM HEPES, pH 7.5, 500 mM NaC)] Superdex 200 10/300 GL Increase column (GE Healthcare) at a flow rate of 0.6 ml/min. Data-analysis was performed with the ASTRA software package (Astra V 5.3.4.20; Wyatt Technology).

### Crystallization of His_6_-MadC

Crystals of the MadC protein were obtained by using commercial screening from Nextal (Qiagen, Hilden, Germany) in 96 well MRC3 plates (Swissci) by mixing 0.1 μl homogenous MadC (12 mg/ml in 50 mM HEPES pH 8) with 0.1 μl reservoir solution and equilibrated against 40 μl reservoir solution at 12°C using the vapor-diffusion sitting-drop method. After one to 2 weeks crystals grew under a condition containing lithium chloride and MPD. This condition was optimized *via* grid screen which resulted in grown together crystals. A seed stock was made of theses crystals and screened against the MPDSuite (Nextal, Qiagen, Hilden) by mixing the protein, the undiluted seed stock and the reservoir in a ratio of 3:2:1. A few new conditions with crystals were obtained between 12 h up to 5 days and directly harvested out of the initial plate. The best crystals grew in 100 mM HEPES pH 7.5, 30% (v/v) MPD and 5% (w/v) PEG 4000 and reached a maximum size of 97 × 85 × 20 μm after 1 day. Crystals were harvested without further cryoprotection and immediately flash frozen in liquid nitrogen. Diffraction data were collected at beamline P13 (DESY, Hamburg, Germany). Crystals belonged to space group P 2_1_ 2_1_ 21 and diffracted to 1.7 Å resolution.

### X-ray – data processing and structure determination

Data sets were collected from a single crystal of His_6_-MadC on beamline P13 at DESY (EMBL, Hamburg, Germany). These data sets were processed using the XDS package (Kabsch, 2010b) and scaled with XSCALE (Kabsch, 2010a). Initial phases were obtained by molecular replacement using the program PHASER (Emsley and Cowtan, 2004) with the crystal structure of the *L. lactis* NisC protein (PDB entry 2G02) as a template (Li et al., 2006). Model building and refinement were performed using COOT (Emsley et al., 2010) and REFMAC5 (Murshudov et al., 2011). Data refinement statistics and model content are summarized in Supplementary Table S1. The atomic coordinates and structure factors have been deposited in the Worldwide Protein Data Bank (PDB; https://www.wwpdb.org/) under the following accession code for the His_6_-MadC (PDB: 8BYK).

### Figure preparation

For figure preparation the following programs were used: PRISM 8 (GraphPad) Version 8.0.2, PEAQ analysis software (Malvern Panalytical) Version 1.41, Powerpoint (Microsoft Office) Version 16.43, Adobe Acrobat Pro Version 11.0.23.

Figures of the crystal structures and the AlphaFold2 models were prepared using the PyMol software suite (www.pymol.org; Schrödinger; Jumper et al., 2021, Mirdita et al., 2022).

## Data availability statement

The datasets presented in this study can be found in online repositories. The names of the repository/repositories and accession number(s) can be found in the article/Supplementary material.

## Author contributions

VK performed purification, ITC experiments, and structure refinement. OS performed MALS-SEC measurement and analysis of the data. MK and AK performed and analyzed the SPR experiment and data. AH and SG performed crystallization and data collection at DESY-Lab in Hamburg. JR performed SAXS-measurements and data analysis. SS analyzed X-ray data and refinement. VK, SS, and LS wrote the manuscript. All authors contributed to the article and approved the submitted version.

## Funding

The Center for Structural studies is funded by the DFG (Grant number 417919780 and INST 208/761-1 FUGG and INST 208/740-1 FUGG to SS). This work was supported by the DFG (grant Schm1279/13-1 to LS and Grant number 417919780 to SS).

## Conflict of interest

The authors declare that the research was conducted in the absence of any commercial or financial relationships that could be construed as a potential conflict of interest.

## Publisher’s note

All claims expressed in this article are solely those of the authors and do not necessarily represent those of their affiliated organizations, or those of the publisher, the editors and the reviewers. Any product that may be evaluated in this article, or claim that may be made by its manufacturer, is not guaranteed or endorsed by the publisher.

## Footnotes

^1^ http://www.embl-hamburg.de/Auto-Rickshaw

^2^ https://www.ccp4.ac.uk

^3^ https://www.phenix-online.org
