# Supplementary material for: The structure of MadC from Clostridium maddingley reveals new insights into class I lanthipeptide cyclases
Source: Front Microbiol. 2023 Jan 18;13:1057217. doi: 10.3389/fmicb.2022.1057217 (PMC9889658; doi:10.3389/fmicb.2022.1057217)
Supplement: Supplementary file 1 [file Data_Sheet_1.docx]

Supplements

**The structure of MadC from *Clostridium maddingley* reveals new insights into class I lanthipeptide cyclases**

C. Vivien Knospe^1^, Michael Kamel^2^, Olivia Spitz^1^, Astrid Hoeppner^3^, Stefanie Galle^3^, Jens Reiners^3^, Alexej Kedrov^2^, Sander H. J. Smits^1,3^ & Lutz Schmitt^1,*^

^1^: Institute of Biochemistry, Heinrich Heine University Düsseldorf

^2^: Synthetic Membrane Systems, Heinrich Heine University Düsseldorf

^3^: Center for Structural Studies, Heinrich Heine University Düsseldorf

*: To whom correspondence should be addressed

Lutz Schmitt

Institute of Biochemistry

Heinrich Heine University Düsseldorf

Universitätsstr. 1

40225 Düsseldorf, Germany

Phone: +49(0)211-81-10773

Fax: +49(0)211-81-15310

Email: [lutz.schmitt@hhu.de](mailto:lutz.schmitt@hhu.de)

**Keywords: lanthipeptide, cyclases, X-ray structure, class I lantibiotic, ITC**


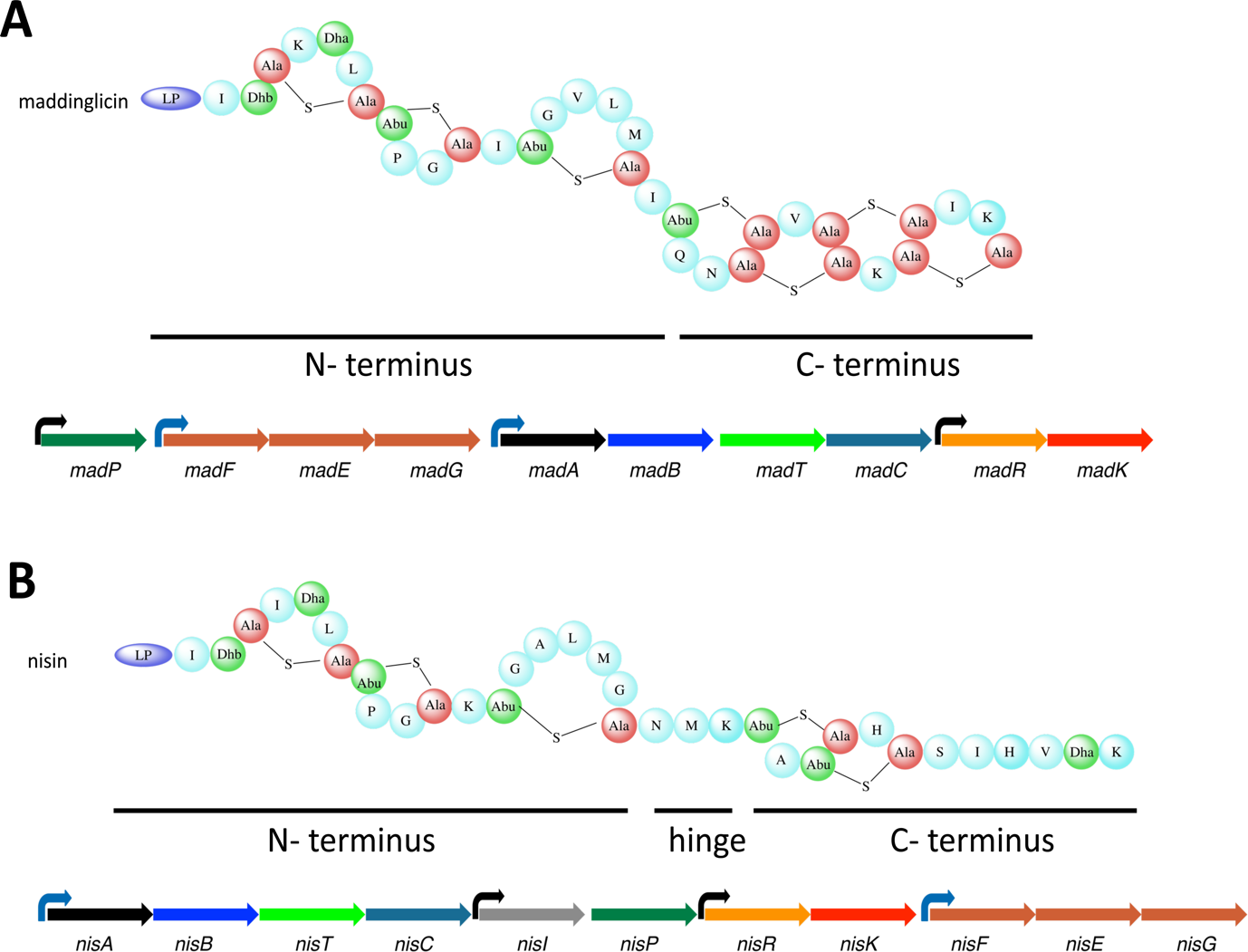


**Figure S1:** Comparison of structures of NisA and MadA and their operon composition. **A:** Predicted structure of Maddinglicin (MadA) obtained via RiPPMiner^1, 2^ and operon of the maddinglicin biosynthetic gene cluster. **B:** Structure of Nisin (NisA) and the Operon of nisin biosynthetic gene cluster. Abbreviations in peptide structures: Ala = alanine; Dha = 2,3-didehydroalanine; Dhb = 2,3-didehydrobutyrine; Abu = α- aminobutyric acid.

**Small-Angle X-ray-Scattering (SAXS) analysis**

We performed Small-Angle-X-ray-Scattering (SAXS) to investigate the homogeneity in solution and to obtain an overall envelop of the protein. We measured a concentration series of 4.4 to 13.8 mg/ml MadC in-house on a Xeuss 2.0 Q-Xoom system at a sample to detector distance of 550 mm. By comparison of the different scattering data, we observed no concentration-dependent effects and continued the evaluation with the highest (13.8 mg/ml) concentration (Supplementary Figure S2, Supplementary Table S2). We determined the radius of gyration (*R_g_*) with the Guinier approximation ^3^ of 2.34 nm, and the maximum particle dimension (*D_max_*) of 7.80 nm with the program GNOM ^4^ (Supplementary Figure S2 B,C). The dimensionless Kratky plot shows a compact globular particle in solution (Supplementary Figure S2 D). Comparison of the theoretical scattering of the MadC crystal structure and the experimental SAXS scattering data offers χ2 of 1.36, indicating a good agreement (Supplementary Figure S2 E).


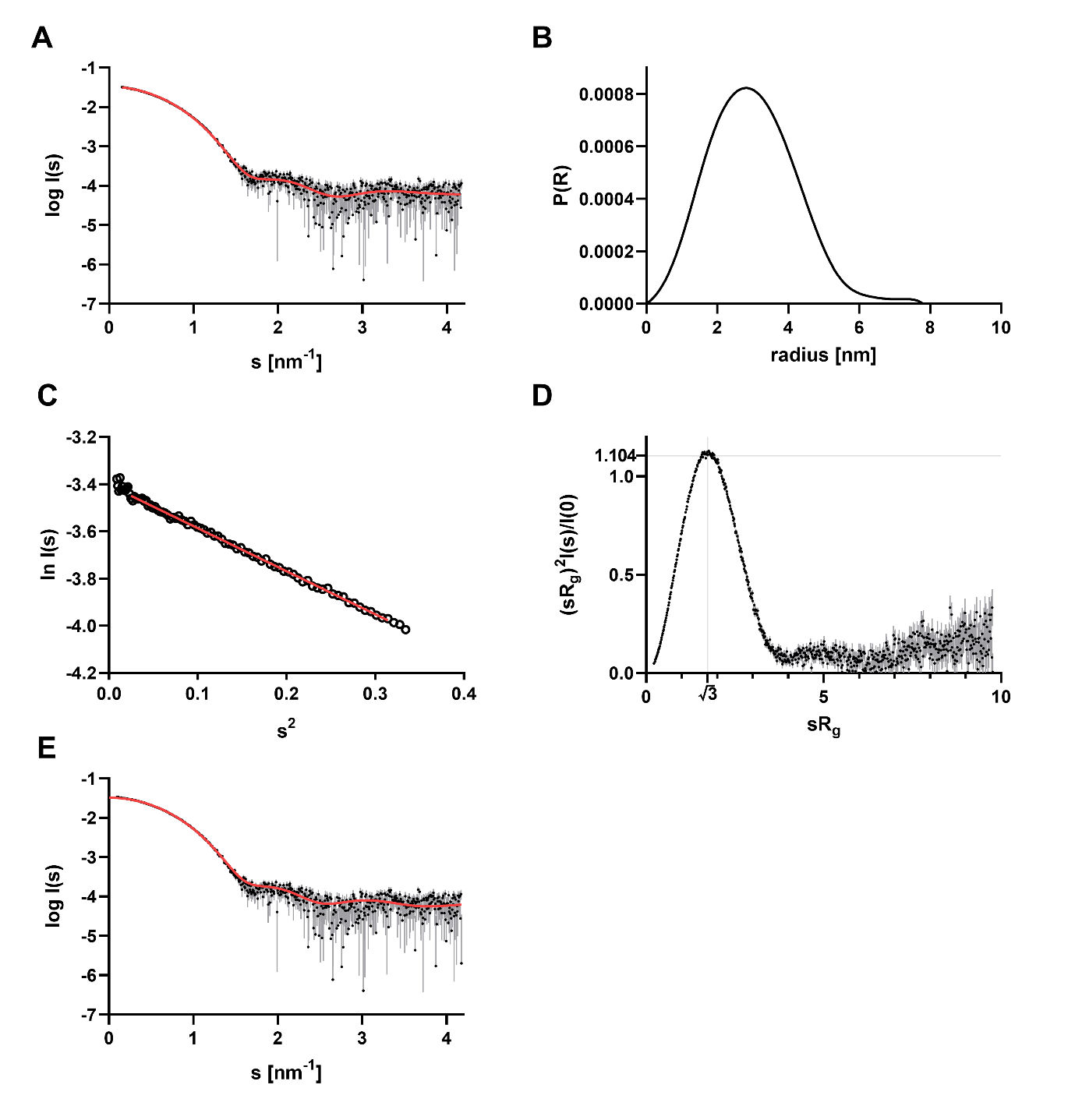


**Figure S2:** Small-angle X-ray scattering data from MadC. **A**: Experimental data curve is shown in black dots with grey error bars. The GASBOR ab-initio model fit (χ2 of 1.26) as red line. The intensity is displayed as a function of momentum transfer s. **B**: The Distance distribution, shown as the *p(r)* function offered a maximum particle diameter (*D_max_*) of 7.80 nm. **C**: Guinier plot of MadC data. A stable Guinier region (red line) was found in the range of sRg < 1.3, with a *Rg* of 2.34 nm. **D**: Dimensionless Kratky plot showed a compact globular particle. **E**: The theoretical intensity of the MadC crystal structure, calculated by Crysol (χ2 of 1.36), showed a good agreement with the experimental data.


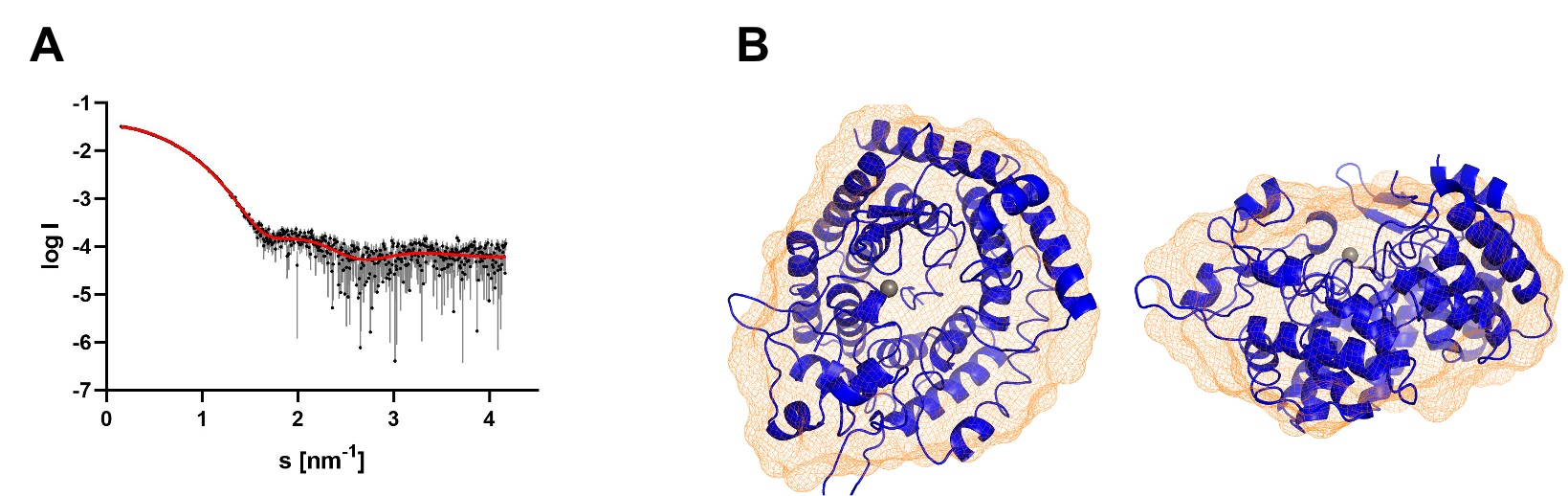


**Figure S3:** Small-angle X-ray scattering data from MadC. **A**: Experimental data curve is shown in black dots with grey error bars. The GASBOR ab-initio model fit (χ2 of 1.26) as red line. The intensity is displayed as a function of momentum transfers. **B**: GASBOR ab initio model of MadC. GASBOR low resolution ab initio model is shown in orange mesh representation Superimposing of the MadC model was done with the program SUPCOMB [6].

**Table S1:** Overall SAXS DATA.

| **SAXS Device** | **Xenocs Xeuss 2.0 with Q-Xoom** |
| --- | --- |
| **Data collection parameters** |  |
| Detector | PILATUS 3 R 300K windowless |
| Detector distance (m) | 0.550 |
| Beam size | 0.8 mm x 0.8 mm |
| Wavelength (nm) | 0.154 |
| Sample environment | Low Noise Flow Cell, 1 mm ø |
| s range (nm^-1^)^‡^ | 0.05 – 6.0 |
| Exposure time per frame (s) | 600 (12 frames) |
| **Sample** | **MadC** |
| Organism | Clostridium sp. Maddingley |
| UniProt ID | K6THC9 (full length) |
| Mode of measurement | batch |
| Temperature (°C) | 10 |
| Protein concentration (mg/ml) | 13.8 |
| Buffer | 50 mM Hepes pH 8.0, 500 mM NaCl |
| **Structural parameters** |  |
| *I*(0) from P(r) | 0.03 |
| *R_g_* (real-space from *P(r)*) (nm) | 2.29 |
| *I*(0) from Guinier fit | 0.03 |
| *s-range* for Guinier fit (nm^-1^) | 0.152 – 0.555 |
| *R_g_* (from Guinier fit) (nm) | 2.34 |
| points from Guinier fit | 11 - 80 |
| *D_max_* (nm) | 7.80 |
| POROD volume estimate (nm^3^) | 78.21 |
| **Molecular mass (kDa)** |  |
| From *I*(0) | 45.70 |
| From Qp ^5^ | 50.86 |
| From MoW2 ^6^ | 54.41 |
| From Vc ^7^ | 44.76 |
| Bayesian Inference ^8^ | 49.76 |
| From POROD | 48.88 |
| From sequence | 49.71 |
| **Structure Evaluation** |  |
| GASBOR fit χ^2^ | 1.26 |
| Ambimeter score | 0 |
| Crysol fit χ^2^ | 1.36 |
| Calculated *R_g_* with Crysol | 2.25 nm (Crystal structure) |
| **Software** |  |
| ATSAS Software Version ^9^ | 3.0.3 |
| Primary data reduction | PRIMUS ^10^ |
| Data processing | GNOM ^4^ |
| Ab initio modelling | GASBOR ^11^ |
| Superimposing | SUPCOMB ^12^ |
| Structure evaluation | AMBIMETER ^13^ / CRYSOL ^14^ |
| Model visualization | PyMOL ^15^ |


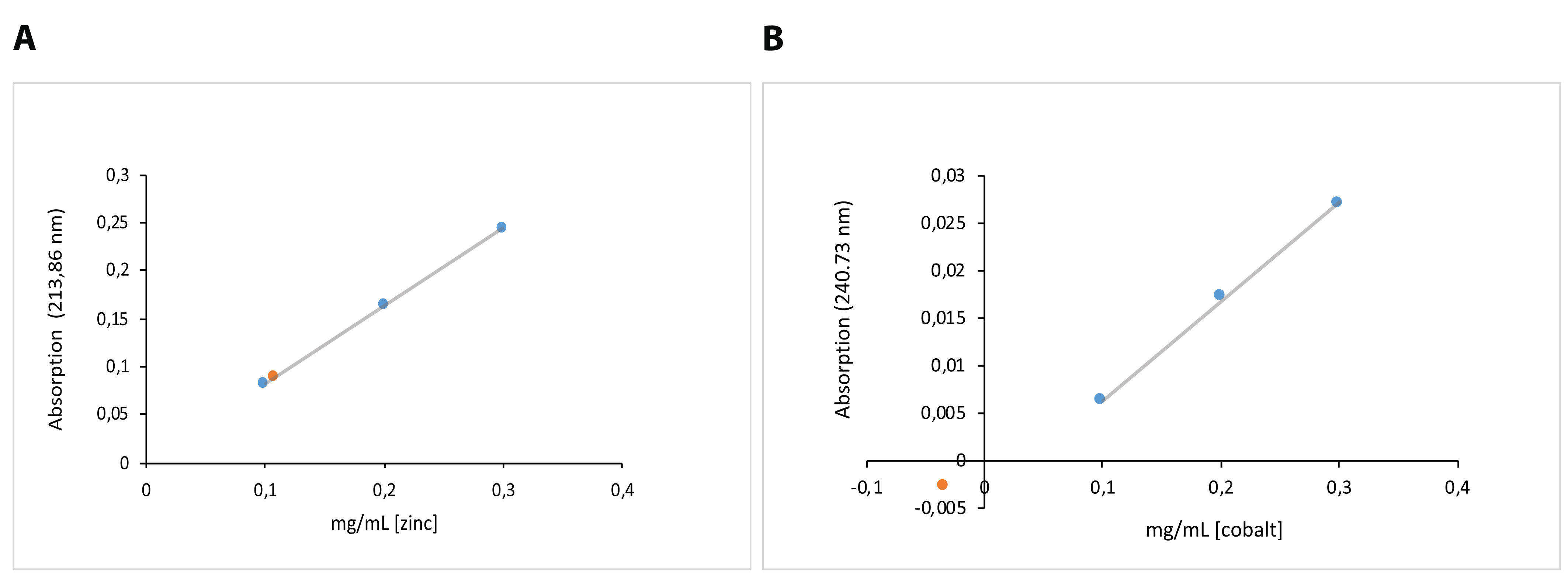


**Figure S4:** Atomic absorption spectroscopy of the MadC sample after IMAC. **A:** Measurement of zinc ion amount in the MadC sample. The blue points indicate the standard measurements for the zinc ion calibration line. The orange point indicates the measured zinc ion amount of the MadC sample. We determined a zinc amount of 0.108 mg/L and expected a concentration of 0.1 mg/L for a stoichiometry of 1:1 for zinc ion per MadC monomer. **B:** Measurement of cobalt ion amount in the MadC sample. The blue points indicate the standard measurements for the cobalt ion calibration line. The orange point indicates the measured cobalt ion amount of the MadC sample.

**Table S2:**  Data collection and refinement statistics of the MadC crystal structure. Number in parentheses correspond to the highest resolution shell (1.761 – 1.7 Å)

|  | MadC |
| --- | --- |
| Wavelength | 0.9763 |
| Resolution range (Å) | 41.89 - 1.7 (1.761 - 1.7) |
| Space group | P 21 21 21 |
| Unit cell (Å) | 54.1 66.1 117.2 90 90 90 |
| Total reflections | 611672 (62541) |
| Unique reflections | 47068 (4591) |
| Multiplicity | 13.0 (13.6) |
| Completeness (%) | 99.97 (99.91) |
| Mean I/sigma(I) | 29.63 (9.90) |
| Wilson B-factor (Å^2^) | 20.06 |
| R-merge (%) | 0.04921 (0.2121) |
| R-meas (%) | 0.05129 (0.2203) |
| R-pim | 0.01428 (0.05929) |
| CC1/2 | 0.999 (0.995) |
| CC* | 1 (0.999) |
| Reflections used in refinement | 47059 (4588) |
| Reflections used for R-free | 735 (72) |
| R-work (%) | 0.1901 (0.2044) |
| R-free (%) | 0.2240 (0.2286) |
| CC(work) | 0.956 (0.954) |
| CC(free) | 0.929 (0.914) |
| Number of non-hydrogen atoms | 3606 |
| macromolecules | 3320 |
| ligands | 1 |
| solvent | 285 |
| Protein residues | 418 |
| RMS(bonds) (Å) | 0.006 |
| RMS(angles) (°) | 0.86 |
| Ramachandran favored (%) | 96.83 |
| Ramachandran allowed (%) | 2.93 |
| Ramachandran outliers (%) | 0.24 |
| Rotamer outliers (%) | 0.00 |
| Clashscore | 7.94 |
| Average B-factor (Å^2^) | 29.90 |
| Macromolecules (Å^2^) | 29.36 |
| Ligands (Å^2^) | 27.94 |
| Solvent (Å^2^) | 36.18 |


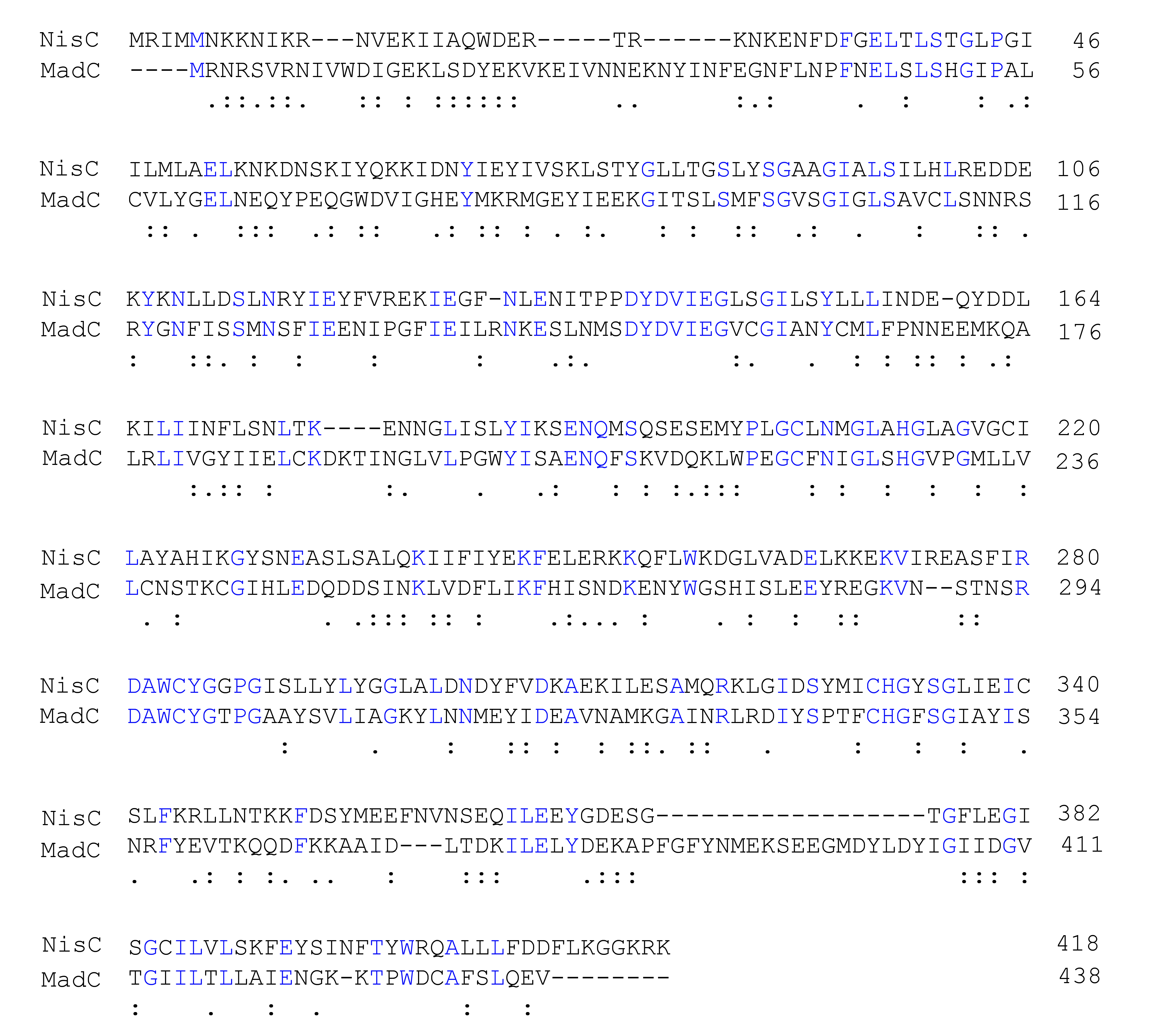


**Figure S5:** Alignment of NisC from L. lactis and MadC from C. maddingley performed by Clustal Omega (Version 1.2.4). (<https://www.ebi.ac.uk/Tools/msa/clustalo/>). Blue colored amino acids indicate identical amino acids for this 2 sequence alignment, “:” indicates very similar amino acids; “.” indicates similar amino acids. See sequences in table S4.


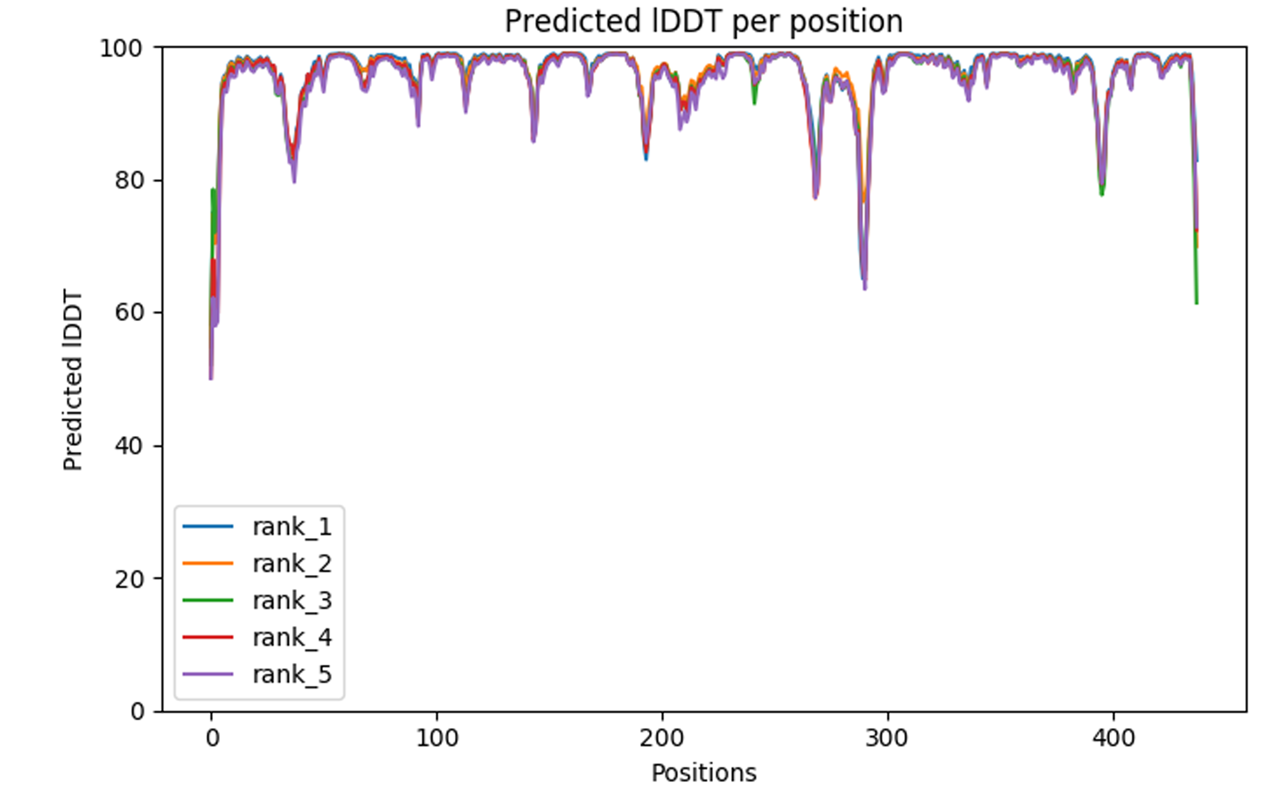


**Figure S6:** Chromatogram of calculated plDDT values for the AlphaFold2 model of MadC. The calculated values for 5 models are colored as shown in the legend.


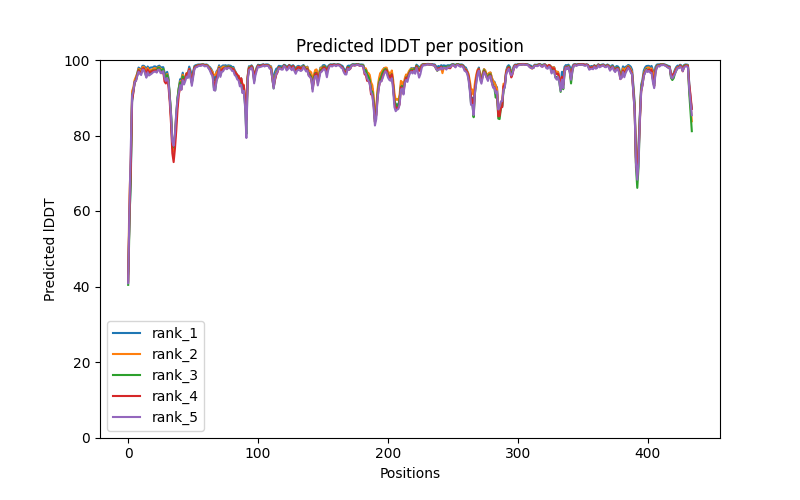


**Figure S7:** Chromatogram of plDDT values for the AlphaFold2 model of GeoC. The calculated values for 5 models are colored as shown in the legend.


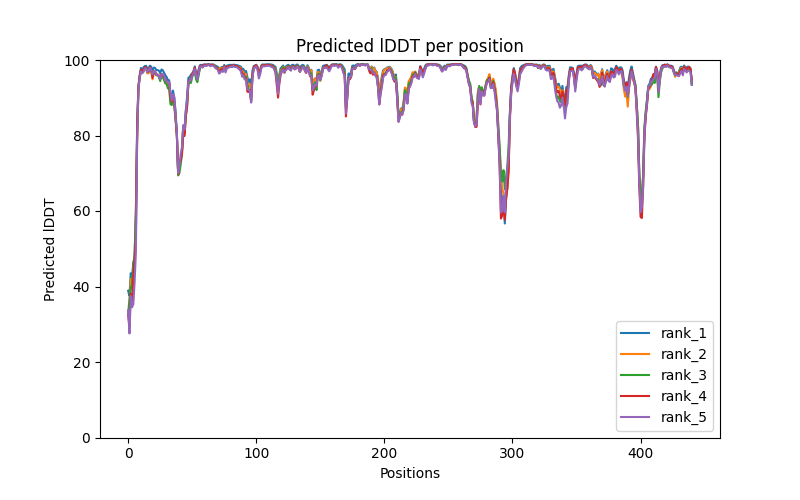


**Figure S8:** Chromatogram of plDDT values for the AlphaFold2 model of SpaC. The calculated values for 5 models are colored as shown in the legend.


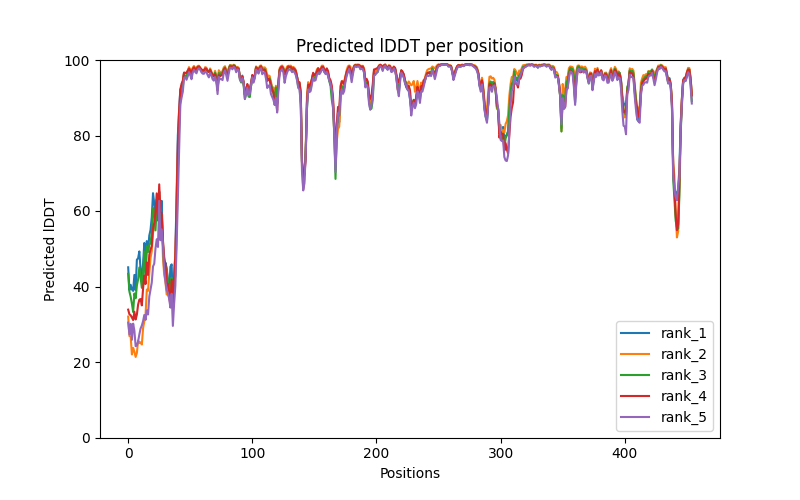


**Figure S9:** Chromatogram of plDDT values for the AlphaFold2 model of EpiC. The calculated values for 5 models are colored as shown in the legend.


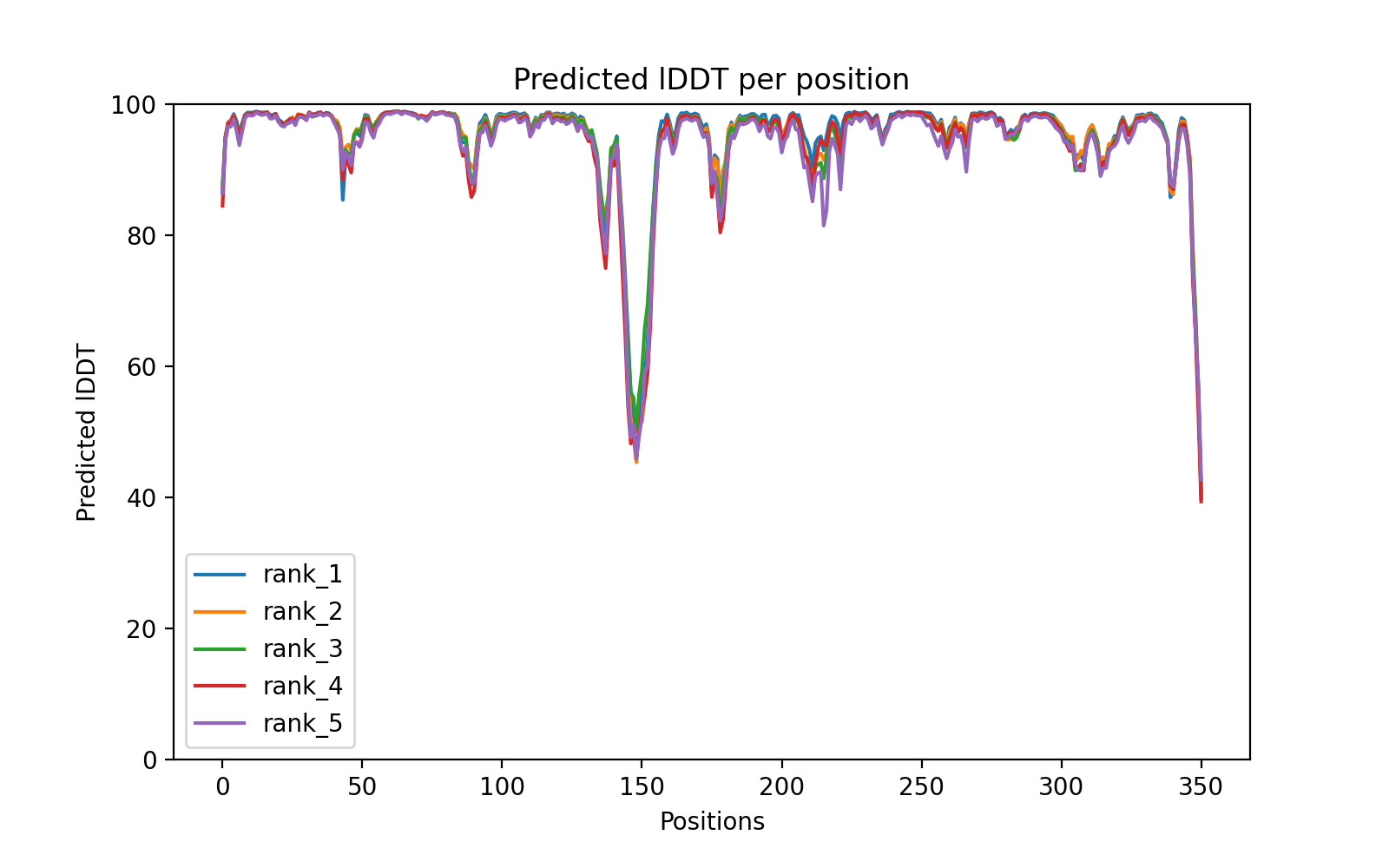


**Figure S10:** Chromatogram of plDDT values for the AlphaFold2 model of SgbL cyclase domain. The calculated values for 5 models are colored as shown in the legend.


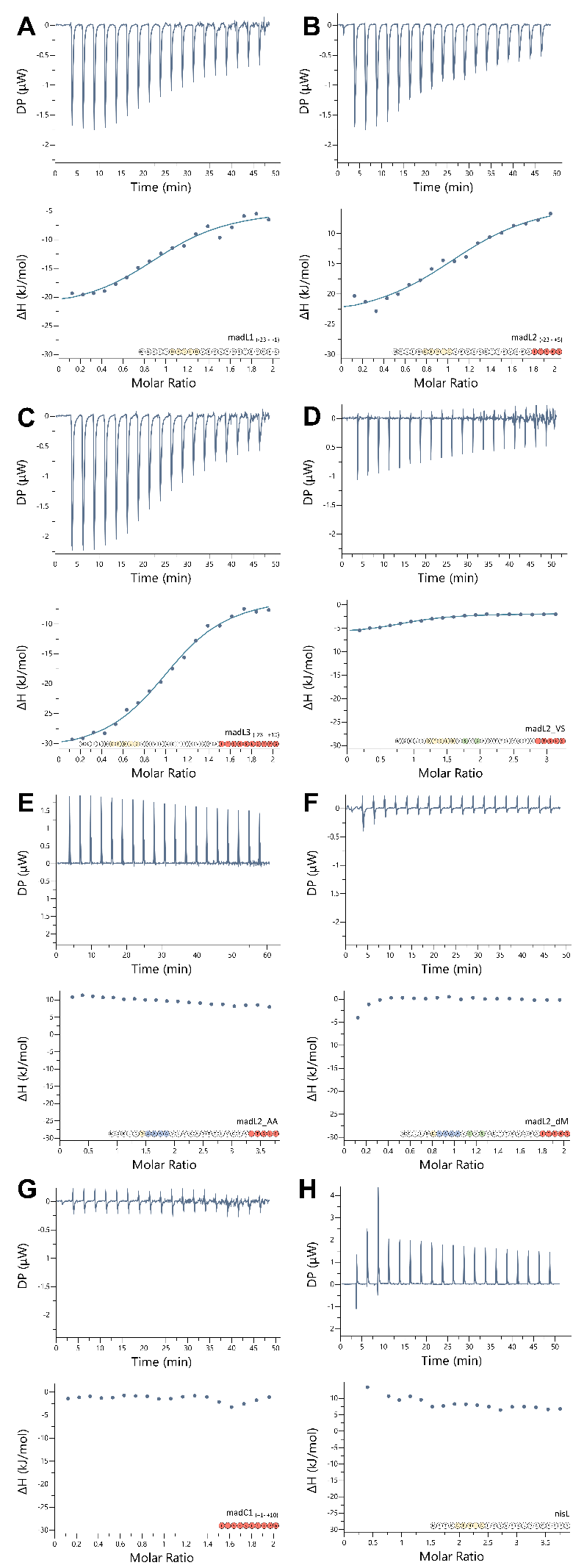


**Figure S11:** ITC experiments of MadC and different maddinglicin leader variants. **A**: maddinglicin leader **B**: maddinglicin leader 2 (L2) [contains 5 aa of the core peptide] **C**: maddinglicin leader 3 (L3) **D**: maddinglicin leader L2_VSVS **E**: L2_AA **F**: L2_dM **G**: core **H**: Nisin leader. For detailed thermodynamic parameters, see Table 1.

**Table S3:** Summary of the used primers in this work and their parameters.

| Primer | Sequence | GC % | Tm |
| --- | --- | --- | --- |
| FOR-pET28b (N.term Gibbs)XhoI | tgactcgagccaccgctgagcaataa | 54% | 64°C |
| REV-pET28b (N.term Gibbs) | catatggctgccgcg | 67% | 57°C |
| FOR-MadC(N.term)NdeI | cgcggcagccatatgatgcgtaatagaagtgttcg | 51% | 68°C |
| REV-MadC(N.term)XhoI | cggtggctcgagtcaaacttcttgtaatgaaaatgcac | 45% | 66°C |
| FOR_MadC∆34-41 | ggagctggtaatccatttaatgaattaagtttatcacat | 31 % | 51°C |
| REV_MadC∆34-41 | ataattcttttcattattaacaatttctttaac | 15% | 52°C |
| FOR_MadC∆391-402 | ggagctggtgattatattggaattattgatggtgtt | 36% | 61°C |
| REV_MadC∆391-402 | ataaaaaccaaatggtgctttttc | 29% | 53°C |


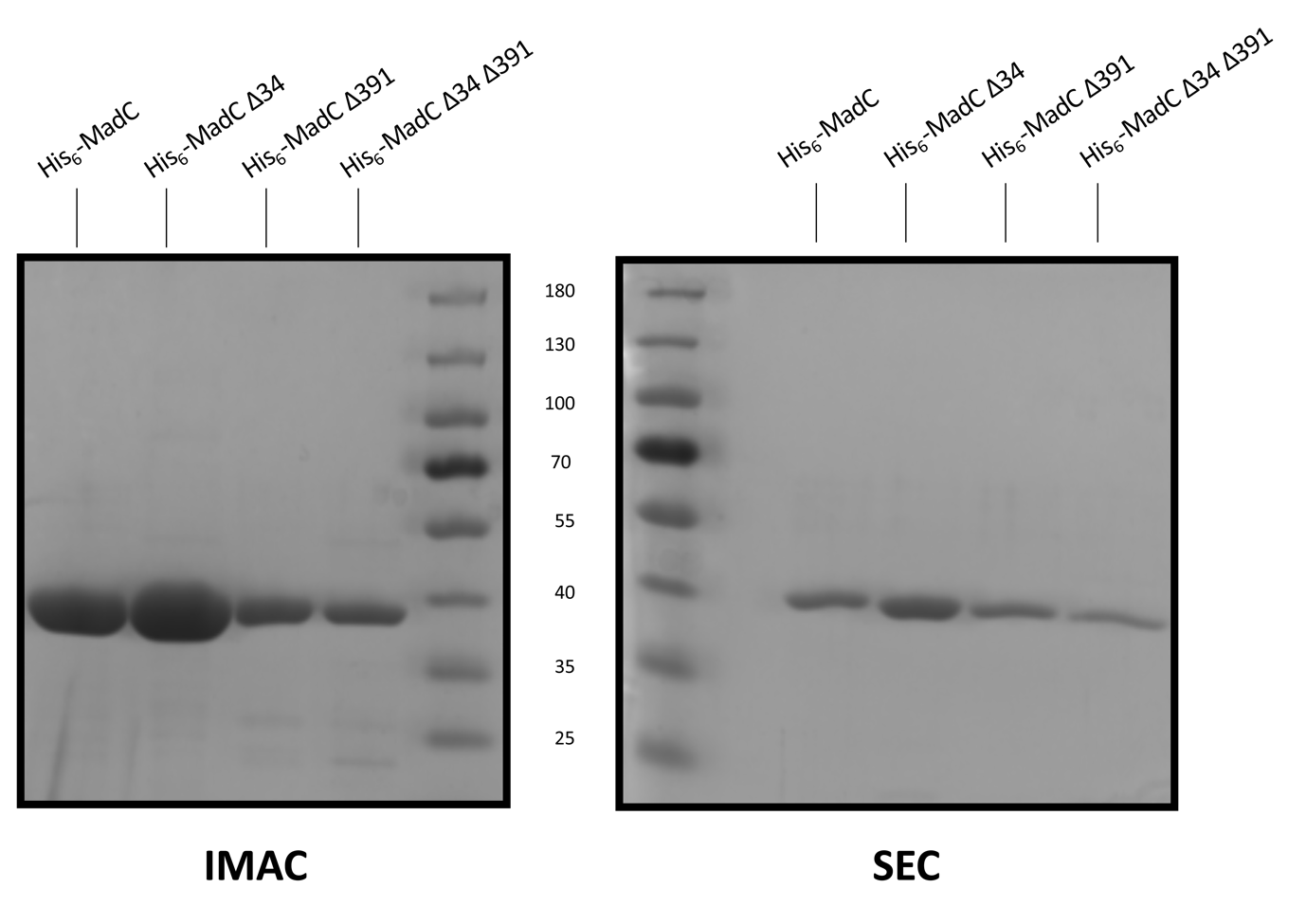


**Figure S12:** Purification of the cyclase MadC from Clostridium maddingley and of the created mutants MadC∆34, MadC∆391 and MadC∆34 ∆391. SDS-PAGE of samples taken during the purification of the MadC variants (left: ion metal affinity chromatography (IMAC), right: size exclusion chromatography (SEC)). Marker: PageRuler Prestained Protein Ladder (Thermo Fisher Scientific), molecular weights in kDa indicated.

**Table S4:** All sequences of LanC enzymes and LanA peptides used in this manuscript for alignments.

| **Protein** | **Sequence** |
| --- | --- |
| MadA (maddinglicin) | >EKQ50563.1 lantibiotic, gallidermin/nisin family [Clostridium sp. Maddingley MBC34-26]  MGKLDDFDLDVKVKADTTKVGPAITSKSLCTPGCITGVLMCITQNSCVSCKSCIKC |
| NisA (nisin) | >sp\|P13068\|LANN_LACLL Lantibiotic nisin-A OS=Lactococcus lactis subsp. lactis OX=1360 GN=spaN PE=1 SV=1  MSTKDFNLDLVSVSKKDSGASPRITSISLCTPGCKTGALMGCNMKTATCHCSIHVSK |
| GeoI (geobacillin) | >ABO65649.1 Lantibiotic antimicrobial precursor peptide [Geobacillus thermodenitrificans NG80-2]  MAKFDDFDLDIVVKKQDDVVQPNVTSKSLCTPGCITGVLMCLTQNSCVSCNSCIRC |
| SpaS (subtilin) | >sp\|P10946\|SPAS_BACIU Lantibiotic subtilin OS=Bacillus subtilis OX=1423 GN=spaS PE=1 SV=1  MSKFDDFDLDVVKVSKQDSKITPQWKSESLCTPGCVTGALQTCFLQTLTCNCKISK |
| EpiA (epidermin) | >sp\|P08136\|LANE_STAEP Lantibiotic epidermin OS=Staphylococcus epidermidis OX=1282 GN=epiA PE=1 SV=1  MEAVKEKNDLFNLDVKVNAKESNDSGAEPRIASKFICTPGCAKTGSFNSYCC |
| PepA /Pep5 | >sp\|P19578\|LANP_STAEP Lantibiotic Pep5 OS=Staphylococcus epidermidis OX=1282 GN=pepA PE=1 SV=1  MKNNKNLFDLEIKKETSQNTDELEPQTAGPAIRASVKQCQKTLKATRLFTVSCKGKNGCK |
| MadC  (C. maddingley) | >EKQ50560.1 Lanthionine synthetase C-like protein [Clostridium sp. Maddingley MBC34-26]  MRNRSVRNIVWDIGEKLSDYEKVKEIVNNEKNYINFEGNFLNPFNELSLSHGIP  ALCVLYGENEQYPEQGWDVIGHEYMKRMGEYIEEKGITSLSMFSGVSGIGLSA  VCLSNNRSRYGNFISSMNSFIEENIPGFIEILRNKESLNMSDYDVIEGVCGIANYC  MLFPNNEEMKQALRLIVGYIIELCKDKTINGLVLPGWYISAENQFSKVDQKLWP  EGCFNIGLSHGVPGMLLVLCNSTKCGIHLEDQDDSINKLVDFLIKFHISNDKENY  WGSHISLEEYREGKVNSTNSRDAWCYGTPGAAYSVLIAGKYLNNMEYIDEAVN  AMKGAINRLRDIYSPTFCHGFSGIAYISNRFYEVTKQQDFKKAAIDLTDKILELYD  EKAPFGFYNMEKSEEGMDYLDYIGIIDGVTGIILTLLAIENGKKTPWDCAFSLQEV |
| NisC (L. lactis) | >sp\|Q03202\|NISC_LACLL Nisin biosynthesis protein NisC OS=Lactococcus lactis subsp. Lactis  OX=1360 GN=nisC PE=1 SV=1  MRIMMNKKNIKRNVEKIIAQWDERTRKNKENFDFGELTLSTGLPGIILMLAELKNKDNSK  IYQKKIDNYIEYIVSKLSTYGLLTGSLYSGAAGIALSILHLREDDEKYKNLLDSLNRYIE  YFVREKIEGFNLENITPPDYDVIEGLSGILSYLLLINDEQYDDLKILIINFLSNLTKENN  GLISLYIKSENQMSQSESEMYPLGCLNMGLAHGLAGVGCILAYAHIKGYSNEASLSALQK  IIFIYEKFELERKKQFLWKDGLVADELKKEKVIREASFIRDAWCYGGPGISLLYLYGGLA  LDNDYFVDKAEKILESAMQRKLGIDSYMICHGYSGLIEICSLFKRLLNTKKFDSYMEEFN  VNSEQILEEYGDESGTGFLEGISGCILVLSKFEYSINFTYWRQALLLFDDFLKGGKRK |
| EpiC | >sp\|P30196\|EPIC_STAEP Epidermin biosynthesis protein EpiC OS=Staphylococcus epidermidis  OX=1282 GN=epiC PE=4 SV=2  MAVLYTCVVIEYSVLILKKKNLFYLFLMKLQKLKNIGMVVININNIKKILENKITFLSDI  EKATYIIENQSEYWDPYTLSHGYPGIILFLSASEKVFHKDLEKVIHQYIRKLGPYLESGI  DGFSLFSGLSGIGFALDIASDKQYSYQSILEQIDNLLVQYVFDFLNNDALEVTPTNYDII  QGFSGIGRYLLNRISYNYNAKKALKHILNYFKTIHYSKDNWLVSNEHQFLDIDKQNFPSG  NINLGLAHGILGPLSLTALSKMNGIEIEGHEEFLQDFTSFLLKPEFKNNNEWFDRYDILE  NYIPNYSVRNGWCYGDTGIMNTLLLSGKALNNEGLIKMSKNILINIIDKNNDDLISPTFC  HGLASHLTIIHQANKFFNLSQVSTYIDTIVRKIISHYSEESSFMFQDIEYSYGQKIYKNK  VGILEGELGVLLALLDYIDTQNQSRKNWKNMFLIT |
| PepC | >tr\|Q54124\|Q54124_STAEP PepC OS=Staphylococcus epidermidis  OX=1282 GN=pepC PE=4 SV=1  MNRFLNTYIKQVTNIDNVDSYINNLYGPEPIYKASLITGYPGIAISLFAIYKETNNFEYY  ELCNKYLEKTIELINDTPMYSTSLFEGAFGTIFSLLVCSDSGSNYSNIIKNLLFEYKKIS  KNEIDRLRTKLKNNNIQFYEFDIISGCAGTLSLLLLATDIFPELSELLVDEIVQITSILT  ELVIKFNNDDYLLDTILSNLGYAHGIPGIINTLCNSYKRGYGIIKTKKILEQSIFTLLQN  LKLENGTIYIPNDIESPNDYRDAWCYGLPSVAYTIFNVSSTLKNKSLIELSESLLHQVFL  RSDNATKLISPTLCHGFSGVVMISLLMNNNELSSKYQKKIIQSYIDQIDGLYFDINDPSN  FSKDIGLLNGNAGILLTLLSYDNNKLINIRWFDFMIMS |
| SpaC | >sp\|P33115\|SPAC_BACIU Subtilin biosynthesis protein SpaC  OS=Bacillus subtilis OX=1423 GN=spaC PE=4 SV=1  MERGTVSRIEVEIVKEMARQISNYDKVLEIVNQKDNFRSIGEVPLIPWKSTALSHGIPGI  CMLYGELHAHFPEEGWDDIGHQYLSILVNEIKEKGLHTPSMFSGAAGIGLAAICLSQRFT  YYNGLISDINEYLAETVPQLLTEFDQRQVCMSDYDVIEGVSGIANYLLLFQEDKAMGDLL  IDILKYLVRLTEDIIVDGEKVPGWHIPSQHQFTDIEKKAYPYGNFNMGLAHGIPGPICVL  SSALIQGIKVKGQEAAIEKMANFLLEFSEKEQDSLFWKGIISFEEYQYGSPPNAVNFSRD  AWCYGRPGVCLALVKAGKALQNTELINIGVQNLRYTISDIRGIFSPTICHGYSGIGQILL  AVNLLTGQEYFKEELQEIKQKIMSYYDKDYIFGFHNYESMEGEEAVPLQYVGLLDGAVGV  GLGVLNMELGSKTDWTKALLI |
| GeoC | >WP_011886697.1 lanthionine synthetase C family protein [Geobacillus thermodenitrificans]  MSISMKALVCNLAKKLSDYENMKRIVNHPSNYIKIGNKTINPFNELSLSHGLPALCALY  GELSEQYPDEGWDLLGHQYMKKIGEQMNQHGFPSLSMFTGLAGIGLAAVCLSKGGKR  YQNFISTINQVIEERIGEMIEYLKQKPYPNMDDYDAISGVAGIASYCLLFPQEMKKSITLI  LKYIIDLSQDKQMEDINVPGWYIPPMNAFSDTERRKWPSGFFNIGLSHGIPALLIVLCNA  KKLNIYVHGQDECIQRIADFLMKFQIKDENGSYWGTHVSLEEYKNGSVLNKDTRDAWC  YGTPGVAYSLLIAGKTLNNQSYIDCAVSGMKLASKRLYNIFSPSFCHGLSGVAYICNRFY  EETNISDFKEAACKLVDDIIKFYNEEFPFGFKNIEESEGSTKYYDYVGLIDGTAGILLTILA  IQNSKKTPWDCAFLLSEV |
| SgbL | >WP_030690958.1 class IV lanthionine synthetase LanL [Streptomyces globisporus]  MTSHATEVELEDLLRQALRTTGTGAHWAVEADEMWCRVAPVTGTRREQGWKLHVSAT  AASAPEVLTRALGVLLREKSGFKFARSLEQVSALNSRATPRGNSGKFITVYPRSDAEAAAL  ARDLHAATAGLAGPRILSDQPYAARSLVHYRYGAFVGRRRLSDDGLLVWFIEDPDGNPVE  DKRTGRYAPPPWAVCPFPASVPVAPHGGEATSRPVVLGGRFAVREAIRQTNKGGVYRGSD  TRTGAGVVIKEARPHVEGDASGGDVRDWLRAEARTLEKLKGTGLAPEAVALFEHAGHLFL  AQDEVPGVTLRTWVAEHFRDVGGERYRADALAQVARLVDLVAAAHARGLVLRDFTPGN  VMVRPDGELRLIDLELAVLEDEAALPTRVGTPGFSAPERLADAPVRPTADYYSLGATVCFVL  AGKVPNLLPEEPAGRPAEERLAAWLAACTRPLGLPDGVVDMVLGLMRDDPAERWDPSRAR  EALRKVDPTARPGSADRTAVRRTGSSAVAGPVPEGRTADEVVAGLVDHLVDSMTPADDRL  WPVSTLTGESDPCTVQQGAAGVLAVLTRYFELTGDPRLPGLLSTAGRWIADRTDVRSPRPGL  HFGGRGTAWALYDAGRAVDDRRLVEHALDLALAPPQTTPHHDVTHGTAGGGLAALHLWR  RTGDARFADLAVDAADRLTAAARREPSGVGWAVPAEADSPEGGKRYLGFAHGAAGIGCFLL  AAADLSRQPDHRAMALEVGEGLVADAVRIGEAAQWPAQSGDVPTAPYWCHGAAGIGTFLV  RLWQATGDDRFGDLARGSAHAVAERASRAPLAQCHGLAGNGDFLLDLADATGDPVHRDMA  EELADLLLAEGTRRQGHVVFPNEYGEVSSSWSDGSAGILAFLLRTRHAGPRHWMVEQRG |


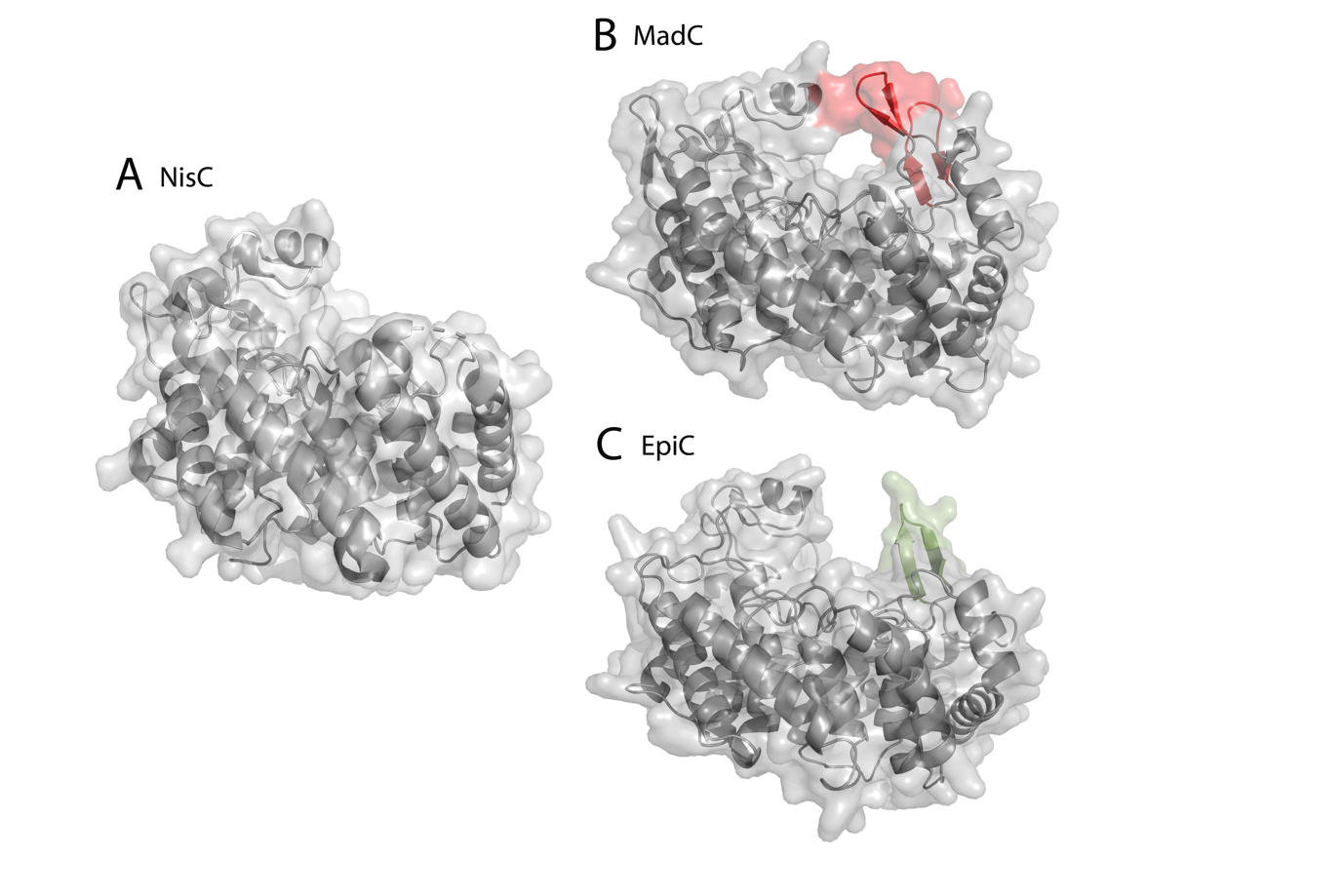


**Figure S13: Overview of the catalytical sites from different LanCs. A**: The crystal structure of the class I cyclase NisC from the nisin system (PDB: 2G02). **B**: The AlphaFold2 model of the class I cyclase MadC of maddinglicin. The β- sheet elements are colored in red. **C**: The AlphaFold2 model of the class I cyclase EpiC of epidermin. The β- sheet element is colored in green. Cartoons and surface were generated in Pymol ([www.pymol.org](http://www.pymol.org)).


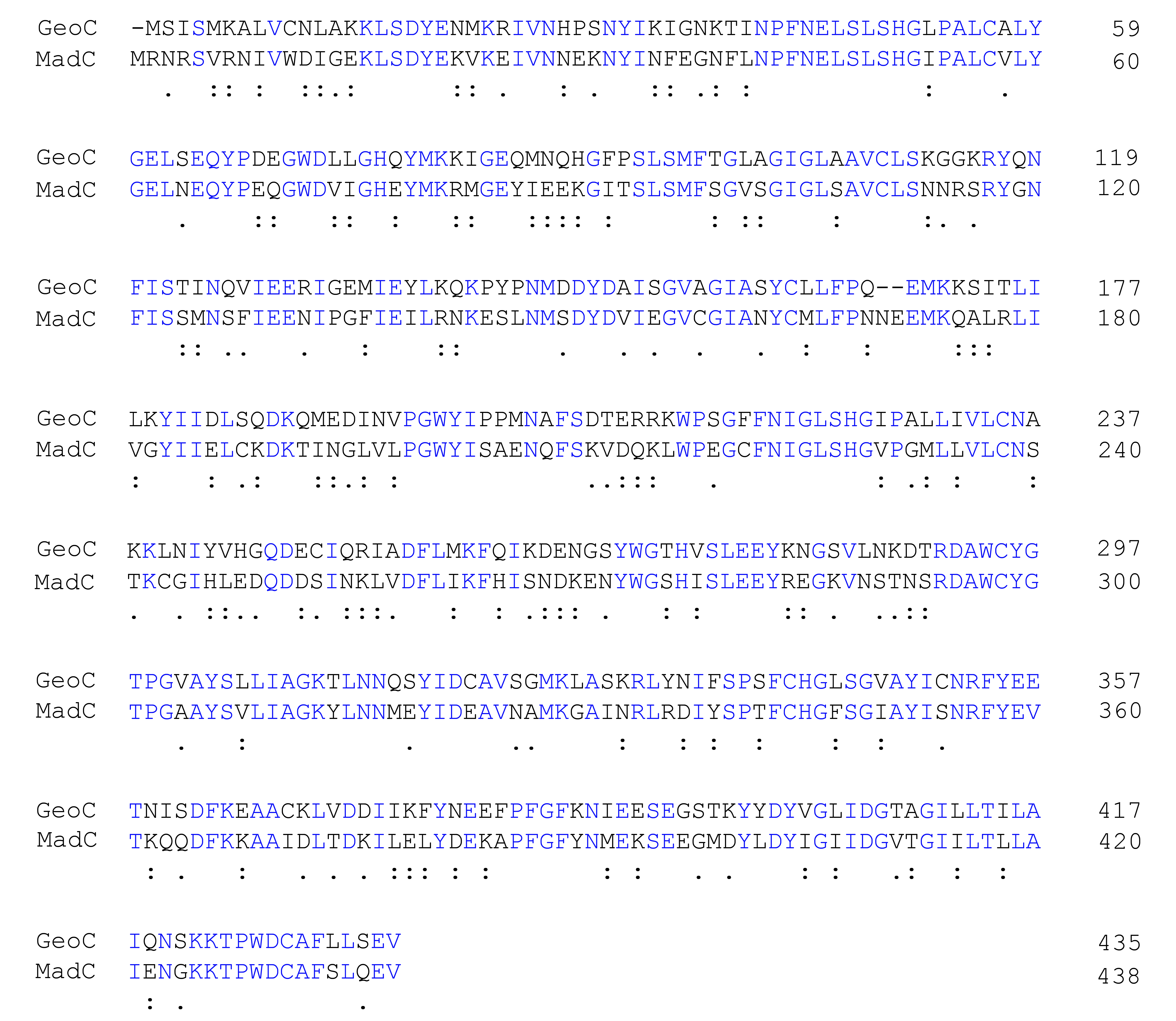


**Figure S14:** Alignment of two class I cyclases performed by Clustal Omega (Version 1.2.4). (<https://www.ebi.ac.uk/Tools/msa/clustalo/>). Sequence alignment of MadC (cyclase of MadA) and GeoC (cyclase of GeoI). Blue colored amino acids indicate identical amino acids for this 2 sequence alignment, “:” indicates very similar amino acids; “.” indicates similar amino acids. See sequences in table S4.





**Figure S15:** Alignment of five class I cyclases performed by Clustal Omega (Version 1.2.4). (<https://www.ebi.ac.uk/Tools/msa/clustalo/>). Sequence alignment of PepC (cyclase of Pep5), EpiC (cyclase of EpiA), NisC (cyclase of NisA), SpaC (cyclase of SpaS) and MadC (cyclase of MadA) via Clustal Omega. Blue colored amino acids indicate identical amino acids for this sequence alignment, “:” indicates very similar amino acids; “.” indicates similar amino acids. See sequences in table S4.

**References:**

1. Agrawal, P.; Amir, S.; Deepak; Barua, D.; Mohanty, D., RiPPMiner-Genome: A Web Resource for Automated Prediction of Crosslinked Chemical Structures of RiPPs by Genome Mining. *J Mol Biol* **2021,** *433* (11), 166887.

2. Agrawal, P.; Khater, S.; Gupta, M.; Sain, N.; Mohanty, D., RiPPMiner: a bioinformatics resource for deciphering chemical structures of RiPPs based on prediction of cleavage and cross-links. *Nucleic Acids Res* **2017,** *45* (W1), W80-W88.

3. Guinier, A., Small-angle X-ray diffraction: application to the study of ultramicroscopic

phenomena. *Annales de Physique* **1939,** *12*, 161-237.

4. Svergun, D. I., Determination of the Regularization Parameter in Indirect-Transform Methods Using Perceptual Criteria. *J Appl Crystallogr* **1992,** *25*, 495-503.

5. Porod, G., Die Röntgenkleinwinkelstreuung Von Dichtgepackten Kolloiden Systemen - 1 Teil. *Kolloid Z Z Polym* **1951,** *124* (2), 83-114.

6. Fischer, H.; Neto, M. D.; Napolitano, H. B.; Polikarpov, I.; Craievich, A. F., Determination of the molecular weight of proteins in solution from a single small-angle X-ray scattering measurement on a relative scale. *J Appl Crystallogr* **2010,** *43*, 101-109.

7. Rambo, R. P.; Tainer, J. A., Accurate assessment of mass, models and resolution by small-angle scattering. *Nature* **2013,** *496* (7446), 477-81.

8. Hajizadeh, N. R.; Franke, D.; Jeffries, C. M.; Svergun, D. I., Consensus Bayesian assessment of protein molecular mass from solution X-ray scattering data. *Sci Rep* **2018,** *8* (1), 7204.

9. Manalastas-Cantos, K.; Konarev, P. V.; Hajizadeh, N. R.; Kikhney, A. G.; Petoukhov, M. V.; Molodenskiy, D. S.; Panjkovich, A.; Mertens, H. D. T.; Gruzinov, A.; Borges, C.; Jeffries, C. M.; Svergun, D. I.; Franke, D., ATSAS 3.0: expanded functionality and new tools for small-angle scattering data analysis. *J Appl Crystallogr* **2021,** *54* (1).

10. Konarev, P. V.; Volkov, V. V.; Sokolova, A. V.; Koch, M. H. J.; Svergun, D. I., PRIMUS: a Windows PC-based system for small-angle scattering data analysis. *J Appl Crystallogr* **2003,** *36*, 1277-1282.

11. Svergun, D. I.; Petoukhov, M. V.; Koch, M. H., Determination of domain structure of proteins from X-ray solution scattering. *Biophysical journal* **2001,** *80* (6), 2946-53.

12. Kozin, M. B.; Svergun, D. I., Automated matching of high- and low-resolution structural models. *J Appl Crystallogr* **2001,** *34*, 33-41.

13. Petoukhov, M. V.; Svergun, D. I., Ambiguity assessment of small-angle scattering curves from monodisperse systems. *Acta Crystallogr D Biol Crystallogr* **2015,** *71* (Pt 5), 1051-8.

14. Svergun, D.; Barberato, C.; Koch, M. H. J., CRYSOL - A program to evaluate x-ray solution scattering of biological macromolecules from atomic coordinates. *J Appl Crystallogr* **1995,** *28*, 768-773.

15. Schrodinger, LLC *The PyMOL Molecular Graphics System, Version 2.0*, 2015.
